# Large language models poorly capture how word order shapes representations of sentence meaning in the human brain

**DOI:** 10.7554/eLife.108442

**Published:** 2026-10-23

**Authors:** James Fodor, Carsten Murawski, Shinsuke Suzuki

**Affiliations:** 1 The Centre for Brain, Mind and Markets, Faculty of Business and Economics, The University of Melbourne Melbourne Australia; 2 Faculty of Social Data Science, Hitotsubashi University Kunitachi Japan; Zhejiang University China; Peking University China

**Keywords:** large language models, representational similarity analysis, fMRI, computational semantics, natural language processing, langauge, Human

## Abstract

Large language models based on the transformer architecture are now capable of producing human-like language. But do they encode and process linguistic meaning in a human-like way? Here, we address this question by analysing 7T fMRI data from 30 participants reading 108 sentences each. These sentences are carefully designed to disentangle sentence structure from word meaning, thereby testing whether transformer representations of sentence meaning resemble those formed by the brain. Using representational similarity analysis, we compared representations from eight transformers and eleven other semantic models to brain activity in language regions of interest. We found that while transformer models match brain representations better than models that completely ignore word order, all transformers poorly match human brain representations of sentences that require sensitivity to structural relations such as semantic roles (who did what to whom). Further, transformers were significantly inferior to models explicitly designed to encode the structural relations between words. Our results provide insight into the nature of sentence representation in the brain, highlighting the critical role of sentence structure. They also cast doubt on the claim that transformers represent sentence meaning similarly to the human brain.

## eLife digest

Large language models (LLMs), such as Claude and ChatGPT, can generate lengthy, grammatically correct responses to a wide range of queries. But does this mean they truly understand language in the same way as humans? Or are they more like sophisticated artificial parrots, combining learned patterns to mimic human language without comprehending its underlying meaning?

On the one hand, LLMs learn by adjusting the weights of connections between artificial neurons in a large neural network, a process that shares some similarities with learning in biological neural networks. On the other hand, LLMs require vastly more input than human infants to develop language abilities. They also learn primarily by predicting the next word based on preceding words, without the social and physical interactions that shape language learning in humans. Given these similarities and differences, the extent to which LLMs process language like humans remains an open question.

To investigate this question, we focused on how LLMs process individual sentences. Specifically, we asked whether they primarily respond to the words themselves or are also sensitive to the order of words and their grammatical roles within a sentence. For example, ‘dog bites man’ and ‘man bites dog’ contain the same words but convey very different meanings. Many previous studies of LLMs have not adequately disentangled the effects of word meaning from those of sentence structure, making it difficult to determine whether these models process sentence structure in a human-like way.

We developed a set of sentences designed to separate the effects of word meaning from those of sentence structure. We then used functional magnetic resonance imaging (fMRI) to measure brain activity in participants as they read these sentences and compared the results with the internal representations generated by LLMs processing the same sentences. We found that LLMs were sensitive to sentence structure, but substantially less so than humans. Human brain activity changed considerably when participants read ‘dog bites man’ rather than ‘man bites dog’. In contrast, the internal representations of LLMs changed much less between the two sentences.

Our findings provide evidence that LLMs process language differently from humans, suggesting that their language processing may not fully reflect human-like understanding. This research has potential applications in two areas. First, insights into differences between human and artificial language processing could inform the development of LLM architectures that more closely resemble human language processing, potentially helping to reduce their unpredictable or unexpected behaviours. Second, a better understanding of how the human brain processes language could help improve treatments for language impairments following stroke or brain surgery. Nevertheless, our research primarily addresses a fundamental scientific question, and further work is needed to determine whether, and to what extent, LLMs truly understand human language.

## Introduction

Understanding how human language is processed and represented in the brain is a major scientific challenge. The past decade has seen a proliferation of work integrating theoretical approaches from linguistics and computer science with empirical data from neuroimaging studies in an effort to better understand how meaning is represented in the brain (Mitchell et al., 2008; Just et al., 2010; Wehbe et al., 2014; Huth et al., 2016; Pereira et al., 2018; Günther et al., 2019; Karamolegkou et al., 2023). Most research has focused on evaluating vector-based models, in which the meaning of a word or phrase is represented as a vector of numbers. This approach forms the basis for large language models, which are neural networks based on the transformer architecture and trained to predict hidden or upcoming tokens on very large corpora of natural text. Leading models such as GPT-4, Gemini, Llama, and Claude are highly versatile, capable of generating grammatical and relevant responses to a wide range of queries and instructions (Ouyang et al., 2022; Touvron et al., 2023; Team et al., 2023). The extensive linguistic capabilities of these models, along with their ability to acquire language competence from naturalistic data, have generated significant interest in their potential value as cognitive models of language processing in humans (Bhatia et al., 2019; Erk, 2022; Tuckute et al., 2024). Studies have consistently found statistically significant correlations between brain activity and various semantic models, with several finding that transformers better explain brain activity compared to static word embedding models (Anderson et al., 2021; Schrimpf et al., 2021; Antonello et al., 2021; Pasquiou et al., 2022).

Most research comparing language models to brain activity has used stimuli that have not been selected to evaluate any specific linguistic hypothesis. While there are many benefits to utilising naturalistic stimuli in the study of language (Aliko et al., 2020; Hamilton and Huth, 2020; Nastase et al., 2021; Zhang et al., 2021), such stimuli have the disadvantage that they may not adequately sample the linguistic phenomena of most interest (Hamilton and Huth, 2020), and do not control for variables crucial for contrasting the representations of different models (Li et al., 2024). A particular challenge is distinguishing whether language models are predictive of brain activity solely due to word-level (lexical) semantic information, or whether they also incorporate representations of sentence structure in a manner comparable to the brain. Direct comparison of static word embeddings with contextualised transformer embeddings is insufficient to resolve this issue, because contextualised embeddings also capture polysemy and other semantic phenomena not directly related to sentence structure. Another limitation of existing studies is that establishing that features extracted from large language models are predictive of brain activity does not necessarily provide much insight about what information these features encode or how such information is utilised by the brain (Kriegeskorte and Douglas, 2019; Bruffaerts et al., 2019; Hagoort, 2020). A final limitation of existing studies is that encoding techniques are best suited to use with vector representations of language, making it difficult to conduct comparisons with graph-based or other approaches specialised for explicitly representing sentence structure.

Here, we present results from an fMRI study in which 30 participants read isolated sentences and answered simple questions about their meaning. We also collected a separate dataset of behavioural ratings of all pairwise comparisons of the same set of sentences. First, we developed a handcrafted set of sentences designed specifically to control for the confound of lexical similarity, allowing for clearer inferences about how sentence-level information (such as semantic role) is represented by the brain. Second, we conduct model comparison using representational similarity analysis (RSA), which involves comparing pairwise similarity scores for voxel activations and semantic models. This technique extracts information about the patterns of similarity of model representations, thereby providing additional insight into the nature of brain semantic representations beyond voxelwise predictability. Furthermore, RSA facilitates comparison between dissimilar types of representations, thereby allowing us to compare a wider range of computational models, including both vector-based and graph-based models, than has been assessed by most previous research (Wang et al., 2018; Fernandino et al., 2022; Tong et al., 2022; Acunzo et al., 2022; Fairhall, 2024).

## Results

### Stimuli and models

**Figure 1.**
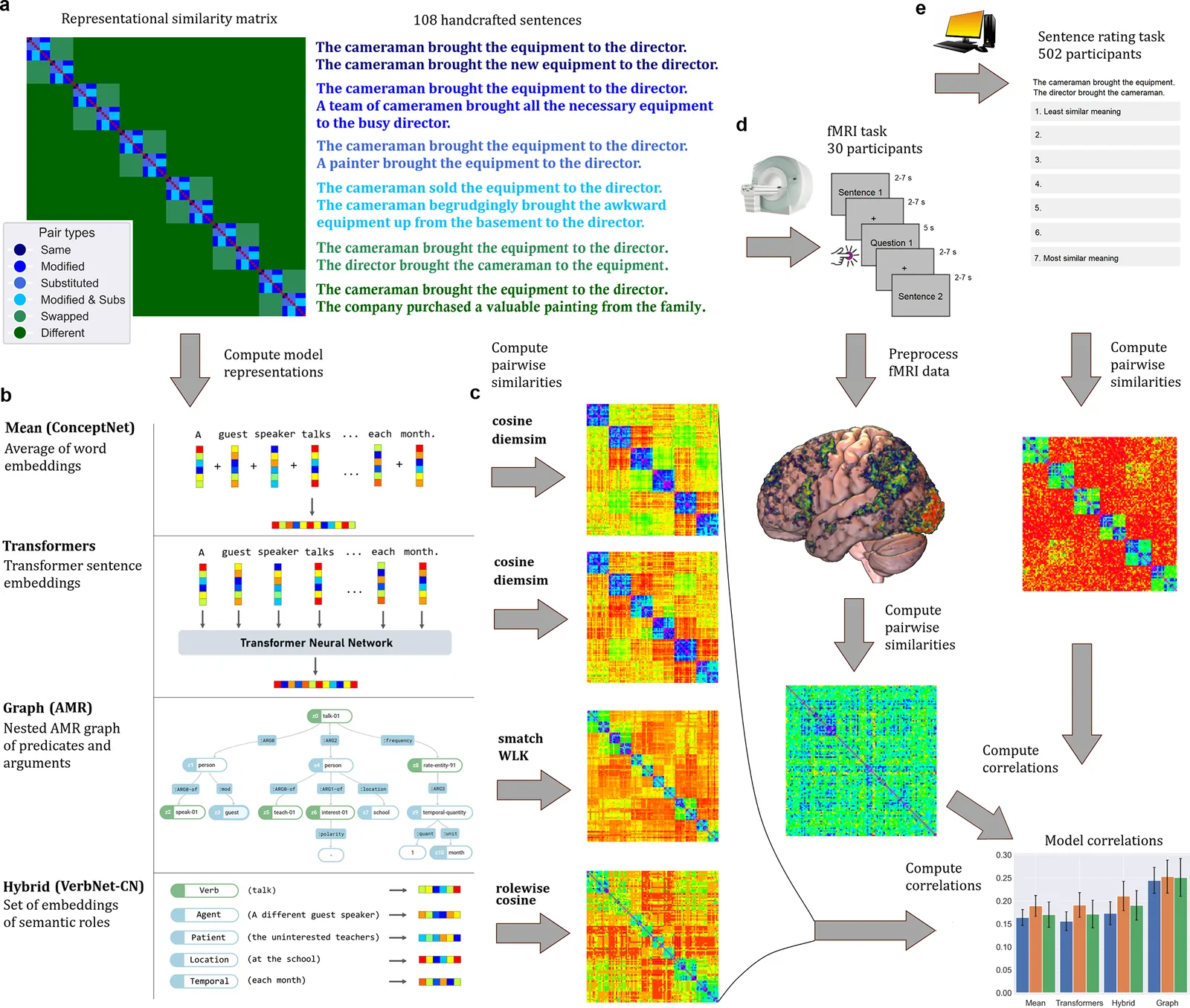
Summary of study methods for constructing stimuli, computing model representations, and collecting fMRI and behavioural data. (**a**) We construct 108 handcrafted sentences, designed to enable systematic variation in sentence meaning while controlling for lexical similarity. Here, we show the corresponding 108×108 design matrix colour-coded with the type of each sentence pair. Sentence pairs in the six blocks along the diagonal are the primary pairs of interest in this study. (**b**) All sentences were encoded using each of the four computational models of sentence meaning which we examine in this study. (**c**) We then computed representational similarity matrices of the 108 stimuli for each of the four models. More similar sentence pairs are shown in blue, and less similar in red. (**d**) Study pipeline for the fMRI experiment, in which participants were presented one sentence at a time for 2–7 s depending on sentence length. Multiple choice comprehension questions were interspersed randomly to assess attention. After scanning, data was processed and brain activity patterns were used to compute a neural representational similarity matrix for each participant. Correlations were then computed between the model and brain representational similarity analysis (RSA) matrices. (**e**) Study pipeline for behavioural experiment, in which online participants were each shown 112 sentence pairs and asked to rate their semantic similarity. Ratings were averaged over participants to compute a similarity matrix. The correlation was then computed between the model and behavioural RSA matrices.

Our handcrafted sentences were carefully designed to reveal the role of sentence structure in semantic representation. Illustrative example sentences are shown in Figure 1a, along with the design matrix indicating the different types of sentence comparisons we considered. This matrix exhibits a block diagonal structure owing to the use of six distinct subsets of sentences, each sharing a similar set of words. Within each of the six subsets, we begin with a base sentence such as ‘the cameraman brought the equipment to the director’, which we then systematically modified in various ways to create different combinations of lexical and compositional similarity, in order to dissociate these two aspects of meaning (see Table 1 for further details). We distinguish between ‘on-diagonal’ and ‘off-diagonal’ sentence pairs. On-diagonal sentence pairs (depicted in shades of blue) have sentence elements simply added or removed. By contrast, the off-diagonal sentence pairs (depicted in light green) have sentence elements interchanged to vary sentence meaning while keeping most of the constituent words the same. This approach builds on our previous work using behavioural data (Fodor et al., 2025), where we showed that such methods allow for effective dissociation of lexical similarity from overall similarity in sentence meaning. We explain the process for constructing the sentences in Methods, Sentence stimuli. The primary objective of the present study is to analyse the brain representations of the block diagonal sentences extracted during an fMRI reading task and compare these to representations derived from a variety of computational models of sentence meaning to determine which models best match brain representations.

**Table 1.** Explanation of the process of constructing sentences used in the study. Added or altered elements in the second sentence in each pair are italicised. The final two columns represent approximate relative similarities intended for each sentence pair type, though there will be variation due to the precise details of each sentence.

Type	Example sentence pair	Lexical similarity	Overall semantic similarity
Same	The cameraman brought the equipment to the director. The cameraman brought the *new* equipment to the director.	Highest	Highest
Modified	The cameraman brought the equipment to the director. The cameraman *begrudgingly* brought the *awkward* equipment *up from the basement* to the director.	Moderate	High
Substituted	The cameraman brought the equipment to the director. The cameraman *sold* the equipment to the director.	Moderate	Moderate
Modified and substituted	The cameraman *sold* the equipment to the director. The cameraman *begrudgingly* brought the *awkward* equipment *up from the basement* to the director.	Low	Moderate
Swapped (simple case)	The cameraman brought the equipment to the director. The *director* brought the *cameraman* to the *equipment*.	High	Moderate
Swapped (with substitution)	The cameraman brought the equipment to the director. The *director sold* the *cameraman’s equipment*.	Moderate	Low
Swapped (with modifiers)	The cameraman brought the equipment to the director. The *renowned director* brought the *impatient cameraman* to the *equipment from his last project*.	Low	Low
Different	The cameraman brought the equipment to the director. The psychologist warned the secretary about the patient.	Lowest	Lowest

We next computed the representations for each sentence using a range of computational models. We analysed four distinct approaches to semantic representation. The first was a simple ‘Mean’ model, consisting of the element-wise averages of static word embeddings of each word in the sentence. Since this model ignores the position of words within a sentence as well as their grammatical role, it serves as a baseline incorporating only lexical information. The second class consists of embeddings extracted from various transformer neural networks. Results for the ‘Transformer’ model are calculated by computing correlations separately for five different transformer models and then taking a simple average of these correlations (details given in Methods, Stimuli and computational models). Results for each individual transformer are presented in Appendix 1—figure 2. Both Mean and Transformer models are vector-based approaches, as they represent the meaning of a sentence with a vector of numbers (Lake and Murphy, 2023). By contrast, ‘Graph’ models are based on a nested graph formalism constructed in accordance with a semantic parsing paradigm. Here, we selected Abstract Meaning Representation (AMR) as a widely used exemplar of this approach to semantic representation (Žabokrtský et al., 2020). Finally, we analysed a ‘Hybrid’ model called ‘VerbNet-CN’, which includes components from both vector-based and graph-based formalisms. Building on our previous work (Fodor et al., 2025), our VerbNet-CN model uses GPT-4 to tag each word based on its semantic role and then constructs a separate vector embedding for each semantic role. All four models are summarised in Figure 1b.

Having constructed the model representations for our sentences, we next computed the similarities between all sentence pairs, using these data to construct RSA matrices for all four computational models. As shown in Figure 1c, the block diagonal structure corresponding to the six sentence subsets is clearly visible. Sentence pairs within these blocks have higher similarity owing to sharing many words in common, as per our design. More importantly, the RSA matrices also illustrate clear differences between how the four models represent sentences. In particular, the ‘swapped’ off-diagonal sentence pairs are accorded high similarities by the Mean-CN model, much lower similarities by the AMR-Smatch and VerbNet-CN models, and intermediate similarities by the Transformer models (OpenAI embeddings shown for illustration). These differences are consistent with our previous findings that transformers are less sensitive to changes in sentence structure than hybrid or graph models. Here, we aim to test which pattern of representational similarities best matches data collected using neuroimaging during a sentence reading task. The full set of RSA matrices for all models is shown in Appendix 1—figure 1.

### fMRI results

To evaluate how well each model describes sentence processing in the brain, we collected fMRI data from 30 participants while reading each of the 108 sentences. Our experimental pipeline is depicted in Figure 1d, with additional details given in Methods. We presented each sentence four times, with randomly interposed questions incorporated as an attention check. Voxel data were analysed using GLMsingle, an algorithm which fits a haemodynamic response (HRF) function to each voxel and then estimates the response of that voxel to each stimulus. We selected a subset of voxels for further analysis based on their stability score, which is computed as the average correlation of voxel activity across repetitions of the same stimulus (Just et al., 2010; Anderson et al., 2017; Just et al., 2017). We analysed stable voxels within two regions of interest (ROIs): the language network (Fedorenko et al., 2024) and the entire cortex less the primary visual cortex (V1–V4). Model fit was assessed using RSA, with higher correlations indicating that the corresponding model represents the set of stimuli more similarly to the brain.

We performed RSA in two different ways. In the simple-average approach, we computed the RSA correlation for each participant separately and then took the average. In contrast, the group-average approach involves first averaging the RSA matrix across participants and then computing the RSA correlation for this group-averaged matrix (Wang et al., 2018; Fernandino et al., 2022; Handjaras et al., 2016). In each case, we computed the Spearman partial correlation across all 5778 sentence pairs and also across the 918 block diagonal sentence pairs, controlling for differences in sentence length. The full set of results for all 21 models tested is shown in Appendix 1—figure 2. Here, we discuss results of the four models of main interest.

**Figure 2.**
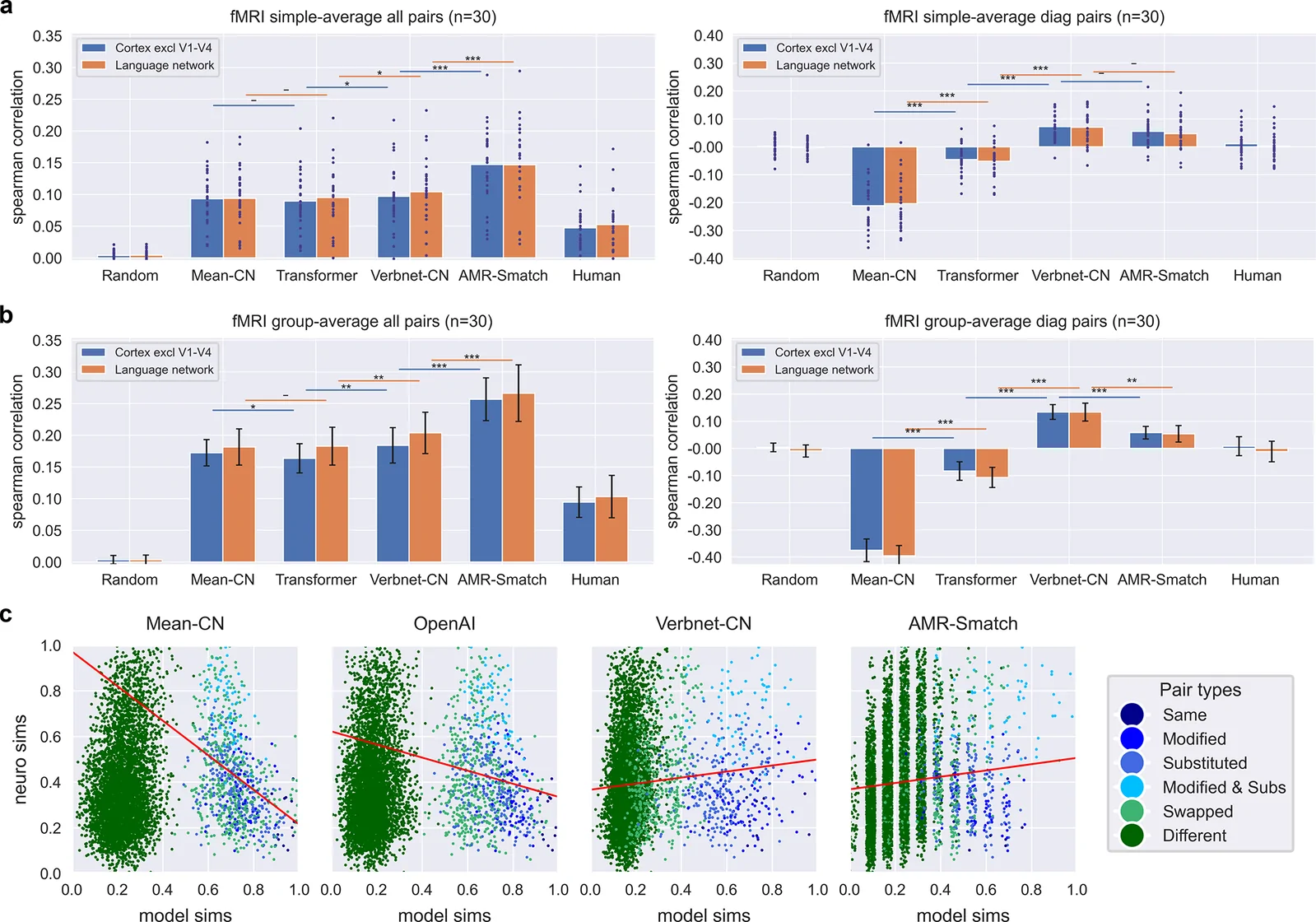
Model correlations with brain activity for all sentence pairs and the block diagonal subset of sentence pairs. Partial correlations between representational similarity analysis (RSA) matrices of five computational models (Random, Mean, Transformer, VerbNet-CN, and AMR-Smatch) and the brain RSA matrix, controlling for differences in sentence length. ‘Human’ refers to behavioural ratings. Blue bars show correlations computed over all stable voxels (excluding visual regions V1–V4), while orange bars show correlations for stable voxels in the language network. Notation for statistical significance: * for p<0.05, ** for p<0.01, and *** for p<0.001, with Bonferroni correction for three independent comparisons. (**a**) Partial correlations for each individual participant shown as blue dots, with the simple average over individual correlations shown as a bar. (**b**) Partial correlations computed using the group-averaged RSA matrix. Error bars show 95% confidence intervals calculated by bootstrap resampling over participants. (**c**) Scatterplots showing the relationship between model similarities (horizontal axis) and group-average neural similarities (vertical axis) for all four computational models. Each dot corresponds to a single pairwise similarity, scaled to between 0 and 1 for visualisation. While all sentence pairs are shown for comparison, regression lines (red) are computed over the block diagonal pairs only.

We first consider correlations computed using all sentence pairs, as shown in Figure 2a. In language network voxels, all models show positive correlations, with relatively small differences between models. For the simple-average method, the differences in correlation were not significant when comparing the Mean and Transformer models ($\Delta \rho =0.001, t=0.686, {\rm p}=0.4981$), and only marginally significant (after multiple comparison correction) for the VerbNet-CN and Transformer models ($\Delta \rho =0.009, t=2.720, {\rm p}=0.0109$). However, the AMR-Smatch model had a significantly higher correlation compared to the VerbNet-CN model ($\Delta \rho =0.043, t=7.393, {\rm p} < 0.0001$). Similar results were found using the group-average method (shown in Figure 2b), but with higher absolute values. The fact that all models show positive correlations when evaluating all sentence pairs is unsurprising, since most sentences can be differentiated from one another using purely lexical differences, which all models are sensitive to.

We now consider correlations computed using only the block diagonal sentence pairs, which are designed to be more difficult for computational models to distinguish owing to high lexical similarity. Here, our results are noticeably different. For the simple average of voxels within the language network, we found a correlation of –0.204 for the Mean-CN model. This comparatively large negative correlation indicates that brain representations of sentences differ systematically from representations constructed considering only lexical similarity, providing evidence that brain representations of sentences are highly sensitive to sentence structure. The Transformer model achieves a correlation of –0.045, which is significantly higher than the Mean-CN model ($\Delta \rho =0.159, t=14.287, {\rm p} < 0.0001$), though the negative sign indicates that transformers still poorly match brain similarities and indeed more closely resemble the static word embeddings that ignore sentence structure. The VerbNet-CN model achieves the highest correlation of 0.070, much larger than the Transformer model ($\Delta \rho =0.115, t=8.150, {\rm p} < 0.0001$). The AMR-Smatch model shows similar results to the VerbNet-CN model, with a correlation of 0.047 ($\Delta \rho =-0.023, t=-1.783, {\rm p}=0.0851$). The correlation with human ratings is very close to zero, placing it between the Transformer and VerbNet-CN models. We consider this surprising result in greater detail in Discussion. Results were very similar using the group-average method, though generally correlations had higher absolute values.

The results for Mean, Transformer, and VerbNet-CN models were all consistent with our preregistered predictions based on previous work with a separate behavioural dataset (Fodor et al., 2025), though we did not make a prediction for the AMR-Smatch model. In all cases, results are very similar whether computed over the entire cortex (excluding V1–V4) or focusing just on the language network. Results are similar when using the dimension insensitive Euclidean metric (DIEM) similarity metric instead of cosine similarity, though with somewhat lower correlations for certain transformer models (see Appendix 1—figures 13 and 14).

To better understand the origin of such large differences in correlations, we plotted neural similarities against the similarities derived from all four computational models (see Figure 2c). For both the Mean and Transformer models, the blue ‘modified’ and ‘substituted’ sentence pairs are accorded similarities comparable to those of the light green ‘swapped’ sentence pairs. By contrast, the VerbNet-CN and AMR-Smatch models generally accord ‘swapped’ sentence pairs distinctly lower similarity than ‘substituted' and ‘modified' sentence pairs. This is easiest to see in the VerbNet-CN subplot of Figure 2c, where the ‘swapped’ sentence pairs are noticeably to the left of the ‘modified' and ‘substituted’ sentence pairs. Such a difference indicates that the VerbNet-CN and AMR-Smatch models have a greater ability to discriminate sentence pairs that are lexically similar but structurally different (due to interchanged semantic roles). This leads to sentence similarities which are in better accord with brain similarity data, thereby driving the positive RSA correlations. These results indicate that when keeping lexical similarity roughly constant, as is the case for the block diagonal sentence pairs, brain similarity patterns are best explained by models that explicitly represent sentence structural elements, namely the VerbNet-CN and AMR-Smatch models. The Mean-CN model, which completely ignores such structure, explains brain representations the worst, with Transformer models doing better than the Mean but still poorly overall.

**Figure 3.**
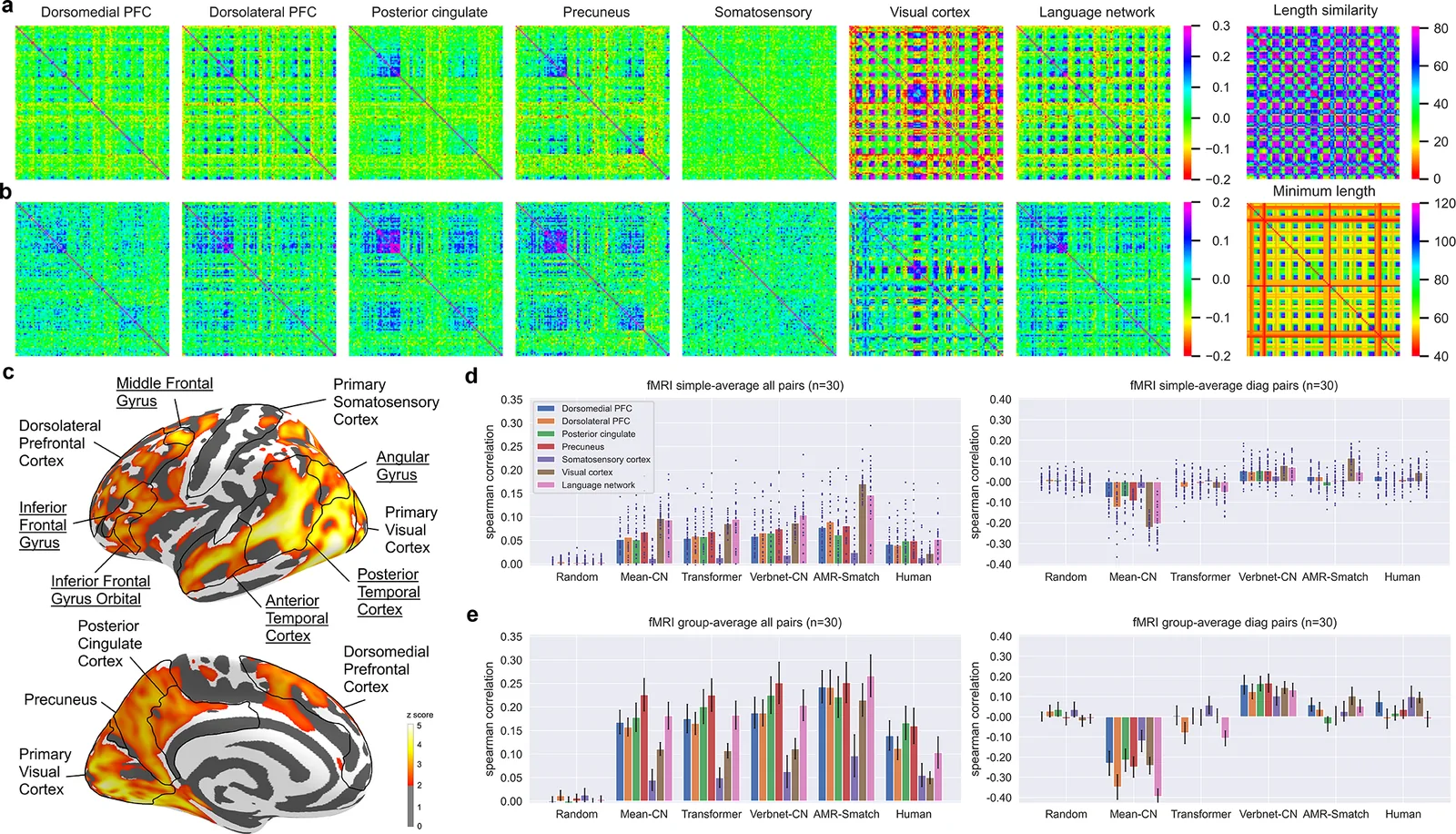
Comparison of sentence representations and model correlations across brain regions. (**a**) Representational similarity analysis (RSA) matrices for various cortical regions, computed controlling for differences in sentence length. (**b**) RSA matrices for various cortical regions, computed controlling for differences in sentence length and minimum sentence length. (**c**) Searchlight RSA for the VerbNet-CN model using 8 mm radius showing cortical regions of interest, with those forming part of the language network underlined. RSA correlations are thresholded at z=2. (**d**) Partial correlations controlling for differences in sentence length by cortical region, with each individual participant shown as blue dots, and the simple average over individual correlations shown as a bar. (**e**) Partial correlations controlling for differences in sentence length computed using the group-averaged RSA matrix, shown by cortical region. Error bars show 95% confidence intervals calculated by bootstrap resampling over participants.

We next compared representations across different brain regions. In addition to the language network and visual cortex (V1–V4), we also considered several regions previously demonstrated to show activity in response to language stimuli, namely the dorsomedial prefrontal cortex, the dorsolateral prefrontal cortex, the posterior cingulate cortex, and the precuneus. The primary somatosensory cortex (S1) is also included as a comparison of a brain region expected to show little response to linguistic stimuli. As shown in Figure 3a, the RSA matrices for most of these regions show a very robust grid-like pattern not explained by the type of sentence pair in the design matrix. This effect is not explained by differences in sentence length, as the RSA matrices already control for this variable (shown in the right of Figure 3a). Upon further investigation, we identified the grid-like pattern as resulting from consistently high brain similarity of sentence pairs in which both sentences are relatively long, as measured by the number of characters. This is evident by visual comparison with the ‘minimum length’ RSA matrix on the right of Figure 3b, which shows the shortest length of the two sentences in each pair. In Appendix 1—figure 4, we show that our main results are qualitatively similar when additionally controlling for the ‘minimum sentence length’ effect. After regressing out this effect using the minimum sentence length for each sentence pair (Figure 3b), we recovered a block diagonal structure comparable to the original design matrix shown in Figure 1a, most clearly visible in the language network. As an additional check, we computed correlations controlling for the fMRI similarities computed over the visual cortex (V1–V4) averaged over all participants. Even with this very strict control of visual similarity, we still observe the same pattern of similarities across the four models (see Appendix 1—figure 9).

**Figure 4.**
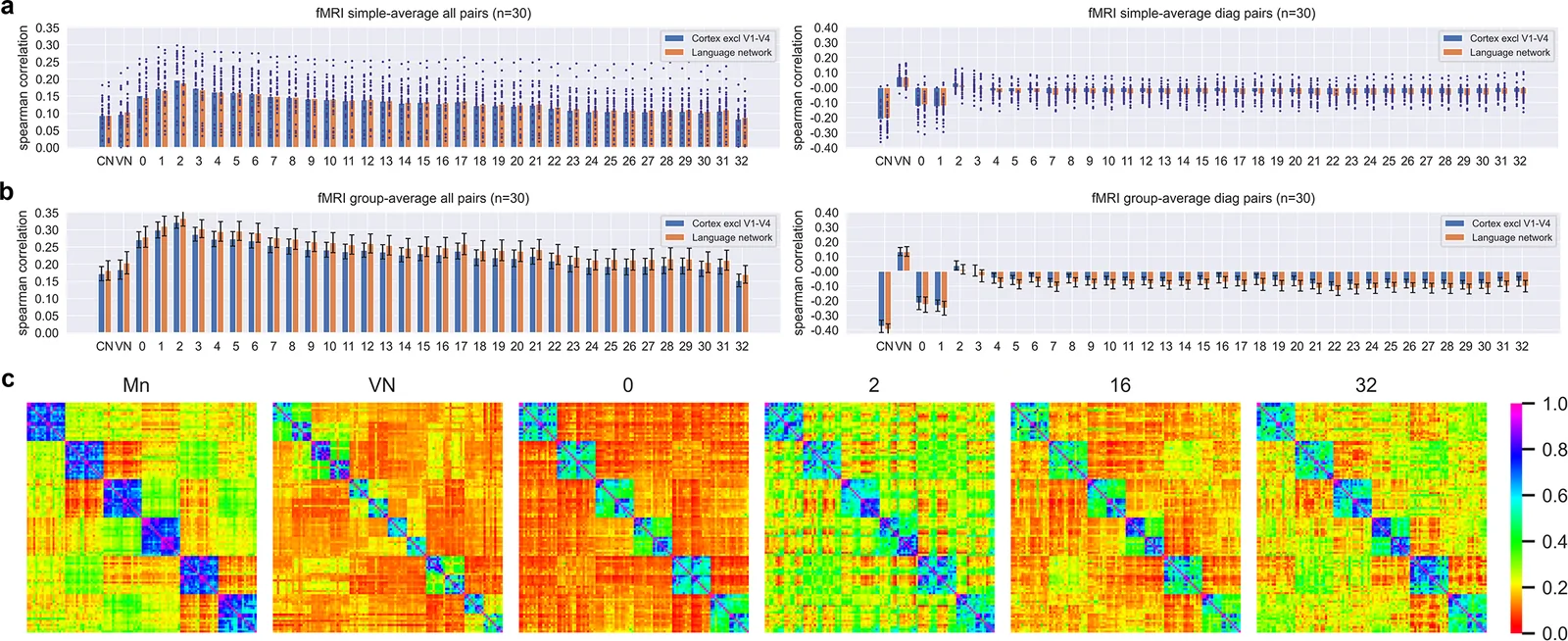
Correlations between individual fMRI data and Llama 3 layers. Average correlations between representational similarity analysis (RSA) matrices of each layer of Llama 3 and brain RSA matrix of each participant. Mean-CN (CN) and VerbNet-CN hybrid (VN) models are also shown for comparison. (**a**) Partial correlations for each individual participant shown as blue dots, with the simple average over individual correlations shown as a bar. (**b**) Partial correlations computed using the group-averaged RSA matrix. Error bars show 95% confidence intervals calculated by bootstrap resampling over participants. (**c**) RSA matrices for the Mean-CN model along with selected layers of the Llama 3 model, computed controlling for differences in sentence length.

We also conducted an analysis of RSA correlations for each layer of the Llama 3 transformer model. We chose this for analysis as a larger, more recent architecture with a large number of layers. As shown in Figure 4, layers 0 and 1 had large negative correlations more similar to the Mean-CN model, while layers 2 and 3 had slightly positive correlations closer to that of the VerbNet-CN model. Layer 4 onwards had more moderate negative correlations, with a slight downward trend over later layers. This pattern was largely similar for both the set of all pairwise comparisons and the set of block diagonal comparison pairs, though in the latter case correlations remained essentially constant from around layer 4 onwards. The corresponding RSA matrices (see Figure 4c) show clear differences in representation across layers, with the earlier layers in particular showing evidence of representations dominated by the effects of sentence length and visual similarity (compare with RSA matrices for Length-sim, Length-min, and Visual in Appendix 1—figure 1). This is dramatically evident when controlling for visual similarities, as this results in correlations over all sentence pairs falling significantly below that of VerbNet-CN, with the highest correlations now observed around layer 28 instead of layer 2 (see Appendix 1—figure 12). We found only modest differences across layers of the AMRBart and ERNIE transformers (see Appendix 1—figures 17 and 18).

To more clearly visualise the location of the brain regions responsible for encoding sentence information in common with the computational models, we conducted an RSA-searchlight analysis. This involves computing the RSA correlation between each model and the voxel activations within an 8 mm sphere surrounding each voxel within a cortical mask. The results (see Figure 3c) show significant correlations throughout the language network, including regions of the temporal lobe, the angular gyrus, and the frontal lobe. Significant correlations are also evident in the posterior cingulate cortex, precuneus, and the visual cortex, with sporadic pockets throughout the dorsolateral and dorsomedial frontal cortical regions. In Figure 3d and e, we show the correlations for each model in each cortical region. We observe low correlations for the somatosensory cortex, generally high correlations for the language network, and intermediate correlations for all other regions. For block diagonal sentence pairs, the VerbNet-CN model has similar correlations across all regions, while the AMR-Smatch model has the highest correlation in the visual cortex, but still positive correlations in the language network. We find similar results when additionally controlling for minimum sentence length, as shown in Appendix 1—figure 5.

**Figure 5.**
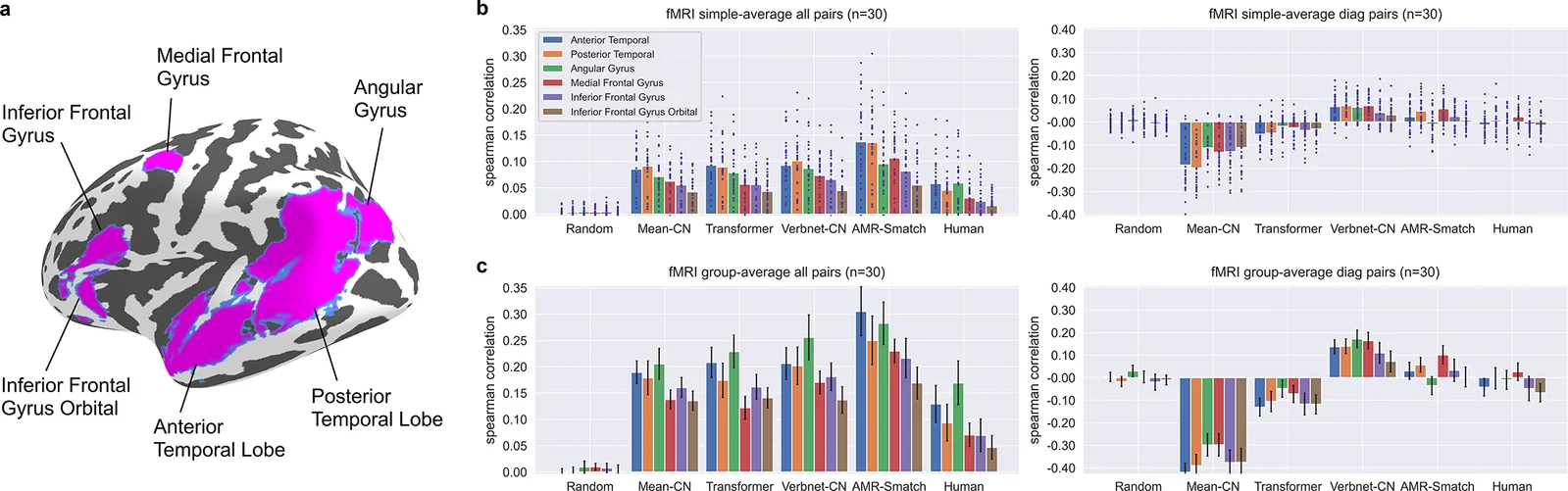
Comparison of model correlations across subregions of the language network. (**a**) Regions within the language network. (**b**) Partial correlations controlling for differences in sentence length by brain region, with each individual participant shown as blue dots, and the simple average over individual correlations shown as a bar. (**c**) Partial correlations controlling for differences in sentence length computed using the group-averaged representational similarity analysis (RSA) matrix, shown by language network region. Error bars show 95% confidence intervals calculated by bootstrap resampling over participants.

We also performed an analysis comparing the representation of each subregion of the language network, the locations of which are depicted in Figure 5a. We found a similar overall pattern of results within all subregions, with consistently positive correlations for the entire set of pairwise comparisons. The magnitude of the correlations varied across subregions, with the highest values observed for the anterior and posterior temporal lobe and lower values for all frontal regions (see Figure 5b and c). For the set of block diagonal sentence pairs, all subregions showed the same pattern as our main results, with a negative correlation for the mean model, modest negative correlations for transformer models, and positive correlations for the hybrid model. These findings support previous results, indicating that all subregions of the language network are sensitive to lexical, syntactic, and compositional aspects of language, without any obvious specialisation across subregions (Shain et al., 2024; Fedorenko et al., 2020). We find little difference when additionally controlling for minimum sentence length, as shown in Appendix 1—figure 7.

### Behavioural results

To supplement our neuroimaging data, we also collected a set of behavioural data consisting of semantic similarity judgements. As illustrated in Figure 1e, we recruited 502 participants using an online platform, each of whom was presented with a set of 102 sentence pairs selected randomly from all 5778 unique sentence pairs. Participants were asked to rate each sentence pair for semantic similarity on a scale of 1–7. Ratings were averaged over participants and scaled to between 0 and 1 for comparison with model similarities. The normalised human sentence similarity ratings ranged from 0 to 0.962, with mean = 0.484 and SD = 0.171 for block diagonal sentence pairs, and mean = 0.072 and SD = 0.071 for all other sentence pairs. The average standard deviation of similarity scores for each sentence pair computed across participants was equal to 0.244 for block diagonal sentence pairs and 0.106 for all other pairs. This is comparable to the 0.19 adjusted average standard deviation of the SICK sentence similarity dataset (Marelli et al., 2014) and 0.216 for the STS3k dataset (Fodor et al., 2025). The split-half reliability with the Spearman-Brown correction was 0.938 for the entire dataset, 0.954 for the block diagonal sentence pairs and 0.715 for all other pairs, indicating high levels of agreement between participants.

**Figure 6.**
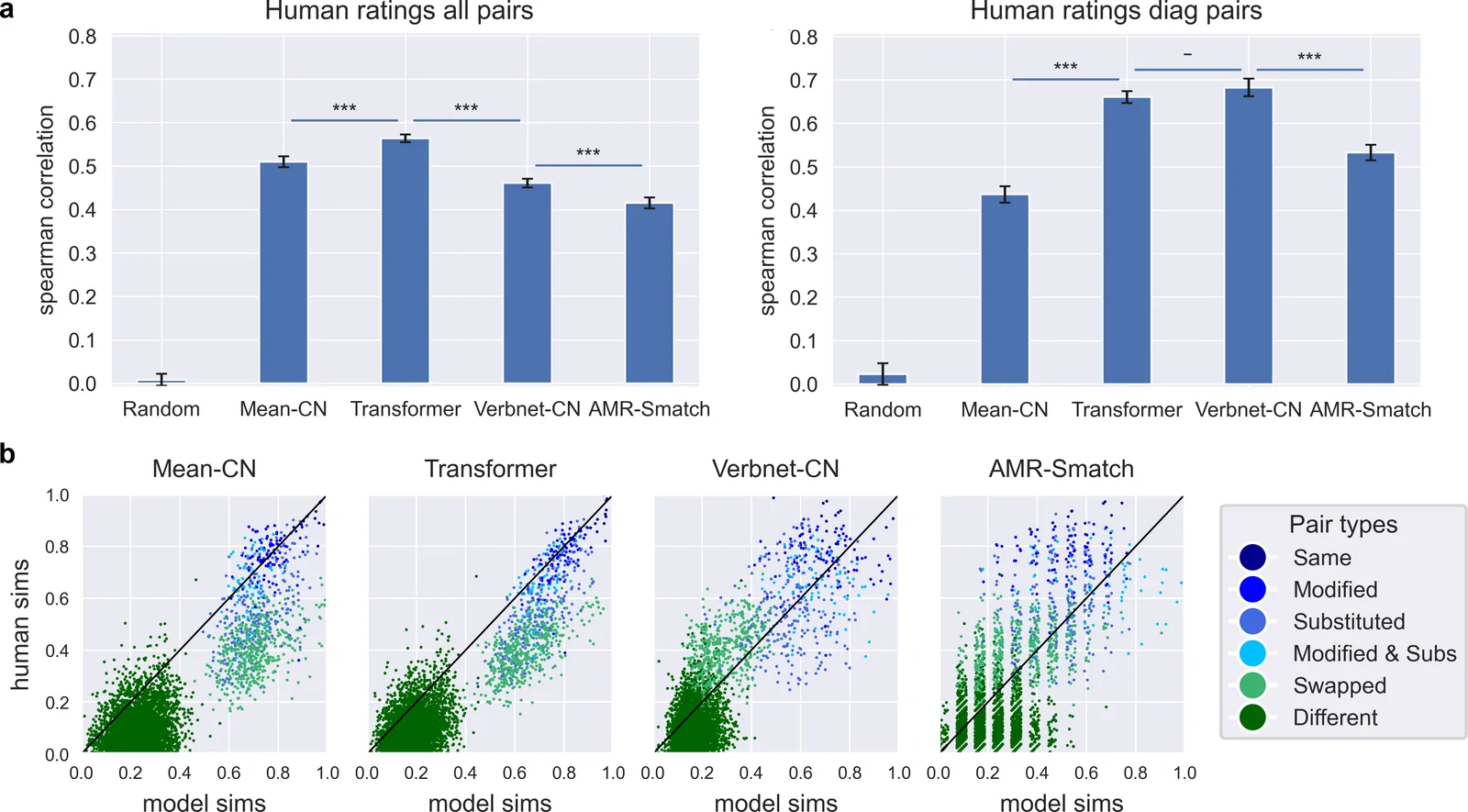
Behavioural ratings of sentence similarity show similar results to fMRI results, but with higher absolute correlations. (**a**) (Left) Average correlations between representational similarity analysis (RSA) matrices of four computational models and human-rated similarities using all sentence pairs. (Right) Average correlations between RSA matrices of four computational models and human-rated similarities using only diagonal sentence pairs. Error bars show standard error of the mean computed over 100 bootstraps. (**b**) Scatterplots showing the relationship between model similarities (horizontal axis) and human rated similarities (vertical axis) for all four computational models. Each dot corresponds to a single pairwise similarity, scaled to between 0 and 1 for visualisation. The 45 degree line (black) shows a hypothetical line of perfect fit between model and human similarities.

We evaluated the fit between behavioural data and each computational model in the same manner as for the fMRI data. For the full set of sentence pairs (Figure 6a, left), the Mean and Transformer models performed best with correlations of 0.510 and 0.564, respectively ($\Delta \rho =0.045,t=9.364, {\rm p} < 0.0001$, computed over bootstrap samples). The VerbNet-CN model had a lower correlation of 0.461 relative to the Transformer model ($\Delta \rho =-0.090,t=-15.450, {\rm p} < 0.0001$), while the AMR-Smatch model had the lowest correlation of 0.416 ($\Delta \rho =-0.046, t=-6.311, {\rm p} < 0.0001$). This pattern was reversed in the case of the block diagonal sentence pairs (Figure 6a, right), with the Mean-CN model having by far the lowest correlation of 0.437. The Transformer model had a much higher correlation of 0.661 ($\Delta \rho =0.210,t=25.054, {\rm p} < 0.0001$), as did the VerbNet-CN model with a correlation of 0.683 ($\Delta \rho =0.024,t=2.261, {\rm p}=0.012$). The AMR-Smatch model had an intermediate correlation of 0.533, lower than the VerbNet-CN model ($\Delta \rho =-0.146,t=-12.939, {\rm p} < 0.0001$). This pattern of results is comparable to that we observed for our fMRI data, though with much higher correlations across all models owing to the much reduced noise in behavioural ratings compared to fMRI voxel data.

As a supplementary analysis not in our original preregistration, we also asked GPT-4 to directly provide ratings for the semantic similarity of each pair of sentences. We found that over both the full set of sentences and the block diagonal subset, these ratings achieved correlations higher than any other method, with values of 0.616 and 0.838, respectively. Correlations for all computational models are shown in Appendix 1—figure 3.

As before, we show scatterplots of the human ratings plotted against model similarities (Figure 6b). While all four models broadly follow the ordering of human ratings along the 45-degree line, both the Mean and OpenAI transformer models place the ‘swapped’ sentence pairs below the line, meaning that these sentence pairs are accorded higher similarity ratings by the models than by humans. By contrast, the VerbNet-CN and AMR-Smatch models place ‘swapped’ sentence pairs above the 45-degree line, meaning that they accord these sentence pairs lower similarities than humans do. These results indicate that for this set of stimuli, the Mean and OpenAI transformer models are less sensitive to variations in sentence structure than human raters, while the VerbNet-CN and AMR-Smatch models are slightly more sensitive to such structure than the human raters.

## Discussion

In this paper, we present, to our knowledge, the first fMRI evaluation of models of sentence representation that utilises stimuli specifically designed to distinguish the effects of lexical semantics from sentence structure. We also present the first quantitative comparison of static word embeddings, transformer neural networks, semantic parsing graphs, and hybrid representational models all under a unified framework. In our neuroimaging experiment, we found that over the block diagonal sentence pairs (the subset of sentence pairs designed to test for sensitivity to sentence structure), considering voxels in the language network, the Mean-CN model had a strong negative correlation, the Transformer model a smaller negative correlation, and the VerbNet-CN model a modest positive correlation. We found similar (though less pronounced) differences in our behavioural experiment. These findings provide two major contributions to our knowledge of sentence representation in the brain. First, we show that controlling for lexical similarity illuminates the brain’s sensitivity to sentence structure in a way that is not evident when the lexical confound is present. Second, the success of our VerbNet-CN model provides novel insight into how sentence structure is represented in the brain, indicating the importance of semantic roles and highlighting limitations of representations derived from transformer models.

### Comparison with previous work

Previous studies analysing sentence processing in the brain have used a variety of controlled stimuli to isolate the mechanisms of semantic composition. One method involves randomly shuffling the order of words within a sentence, thereby preserving lexical semantics while varying overall sentence meaning (Goucha and Friederici, 2015; Mollica et al., 2020). A second method involves constructing ‘jabberwocky’ sentences, which involve nonsensical words placed in grammatically well-formed sentences (Shain et al., 2024; Fedorenko et al., 2012; Matchin et al., 2017). These stimuli are designed to control for syntactic structure or sentence form while manipulating sentence meaning. In both cases, the objective is typically to use jabberwocky or shuffled sentences as a control condition in which composition is prevented, thereby providing a baseline for sentences in which composition occurs (Călinescu et al., 2023). Our study differs from these approaches in that we aim to preserve, rather than prevent, composition. Instead, we control for lexical similarity while constructing semantically meaningful sentences with differing meanings.

Another approach that has seen widespread use is the presentation of minimal sentence pairs that differ only in one specified aspect, e.g., interchanging subject and object in a sentence (Frankland and Greene, 2015; Wang et al., 2016; Frankland and Greene, 2020a; Giglio et al., 2024) or altering adjective-noun phrases to influence composition (Graves et al., 2010; Schell et al., 2017; Fyshe et al., 2019; Ciapparelli et al., 2025). Our approach is an extension of these techniques that utilises more naturalistic and complex sentences, designed to facilitate comparison of a wider range of structural manipulations (see Table 1). By more completely characterising the representational structure of various computational models in response to various semantic contrasts, we are able to more comprehensively evaluate their adequacy as models of semantic processing in the brain.

### Transformer models

Our results indicate that transformer representations do not adequately incorporate sentence structure in a brain-like manner. While most models perform well when evaluated over the full set of sentence pairs, when evaluated against the block diagonal pairs only, transformers are insufficiently sensitive to ‘swapping’ of semantic roles (see Figures 2 and 6), ranking such sentence pairs as more similar than do human participants (in the behavioural data) or brain representations (in the fMRI data). This effect was very robust, with negative correlations observed for all transformers and static word embeddings we studied aside from DefSent (see Appendix 1—figures 2 and 8). These negative correlations result from our experimental design and arise for models which accord high similarities to sentence pairs that are lexically similar, but differ in meaning due to interchanging of semantic roles (see Table 1 for examples). These semantic differences lead to relatively dissimilar brain representations, in contrast to the high similarities of the model representations. The resulting negative correlations therefore indicate that transformer representations are much less sensitive than humans to the effect that semantic roles have on overall sentence meaning.

Similar results were found with the DIEM similarity metric, with negative correlations observed for block diagonal pairs for all transformer and static word embedding models except DefSent and GloVe (see Appendix 1—figures 14 and 15). When visual similarities were controlled for, all transformer and static word embedding correlations fall dramatically, with even DefSent and GloVe becoming negative, indicating that they may be significantly influenced by factors correlated with visual features, such as sentence length or superficial form (see Appendix 1—figure 10, Appendix 1—figure 11, and Appendix 1—figure 16). By contrast, correlations for the AMR-Smatch and VerbNet-CN models are much less affected after introducing controls for visual similarity, with the VerbNet-CN model in particular maintaining a positive correlation over block diagonal sentence pairs for every combination of similarity metric and control condition.

Several previous studies have found that voxelwise encoding models trained using features extracted from transformers are able to better predict brain activity than static word embedding models which ignore sentence structure (Schrimpf et al., 2021; Antonello et al., 2021; Pasquiou et al., 2022; Oota et al., 2024). However, interpreting these findings is difficult because there is no established method for determining which model features drive these correlations (Hadidi et al., 2025). Indeed, some studies have found that even features from untrained transformers can achieve high voxelwise correlations (Schrimpf et al., 2021; Hosseini et al., 2024), casting doubt on whether the transformer features which drive brain correlations are linguistically relevant. Similarly, other studies using shuffled sentences to remove information about sentence structure have found that this results in only modest reductions in voxelwise correlations (Caucheteux et al., 2021; Kauf et al., 2024). An analysis that better controlled for various confounds found that most variance explainable by transformers was accounted for by static word embeddings and word rate (Hadidi et al., 2025). Our results complement these findings, showing that in cases where sentence structure is critical, transformer representations are insufficiently sensitive to structural aspects of sentence meaning. In cases where transformers have been found to have an advantage, this may be due to their greater ability to contextualise polysemous word meanings based on the presence of other words, rather than their ability to represent sentence structure.

We emphasise that our results do not show that transformers fail to represent syntactic or semantic role information. Indeed, large language models show clear capabilities of correctly interpreting sentence structure (Chang and Bergen, 2024), and probing studies have found that transformers represent information about syntax and word order (Clark et al., 2019; Manning et al., 2020). This is consistent with our finding that directly prompting GPT-4 to rate sentence similarity yields very high correlations with human judgements (see Appendix 1—figure 3). Nonetheless, the fact that transformers can encode and utilise structural information to perform linguistic tasks does not mean that they effectively utilise this information to construct a brain-like representation of sentence meaning. Our results indicate that despite the linguistic competencies of transformers, when controlling for lexical similarity, transformers do not combine syntactic and semantic information into an integrated sentence representation in a manner analogous to the human brain. Another problem with using transformers as models of semantic representation is that they are largely ‘black box’ models whose representations are often difficult to understand. Graph-based and hybrid models, whose semantic representations are much more transparent and interpretable, can thus play an important role in increasing our understanding of how semantic information is represented by large language models, and to what extent such representations differ from those formed in the human brain.

### Graph and hybrid models

Our results for the graph-based models were rather mixed. We found that purely syntactic models based on constituency parses (the Benepar and context-free grammar [CFG] models) have low correlations with brain activity (see Appendix 1—figure 2). Examining the corresponding RSA matrices (see Appendix 1—figure 1), this seems to be due to such models being overly sensitive to syntactic form and relatively insensitive to which words are assigned to different nodes within the syntactic tree. This can be seen in the RSA matrices in the four blue squares within each of the six block diagonal squares, which indicates that the ‘swapped’ sentences are not adequately distinguished from ‘same’ sentences (compare with Figure 1a). Comparison with the Length-sim and Length-min RSA matrices also indicates that the edit-distance similarity metrics are strongly affected by sentence length. The AMR-WLK model shows a similar RSA pattern to the Benepar and CFG models, which may account for its low brain and behavioural correlations. Interestingly, the AMR-Smatch model has relatively high brain correlations, despite differing from AMR-WLK only in the similarity metric used. We speculate this could be explained by the fact that SMATCH similarity is based on the number of node triples shared between two graphs, which could be more effective at encoding semantic roles than the more complex node-embedding method used in the WWLK metric (see Methods, Stimuli and computational models for further details). These findings highlight the importance of carefully evaluating graph similarity metrics and identifying which are most appropriate for comparisons of semantic similarity. Several previous studies have likewise emphasised the limitations of existing metrics and the need to explore alternatives (Opitz and Frank, 2022a; Leung et al., 2022; Opitz, 2023).

The Hybrid VerbNet-CN model achieves the highest brain correlations for block diagonal sentence pairs of all models tested, and behavioural correlations comparable to leading transformer models. We believe this is due to this model being designed specifically to be highly sensitive to semantic roles, which is the major point of differentiation from most other models. Indeed, for the behavioural data, we find that the hybrid model is actually more sensitive to these ‘swapped’ sentences compared to human participants, who rate their similarity in between that of the VerbNet-CN and Transformer models (see Figure 6). Interestingly, the second hybrid model we analysed, AMR-CN, shows low brain correlations (see Appendix 1—figure 2). We speculate this is likely due to the crude method in which AMR-CN extracts semantic roles from the uppermost layer of the AMR graph of each sentence, in contrast to the VerbNet-CN model which uses GPT-4 to identify semantic roles directly. Indeed, this difference is why we predicted that VerbNet-CN would perform best in our preregistration. We also wish to emphasise that our core message is not the superiority of the Verbnet-CN model per se, but that the better performance of the VerbNet-CN model over block diagonal sentence pairs highlights that static word embeddings and transformer representations are insufficiently sensitive to semantic roles. These results highlight the value of hybrid approaches designed to appropriately balance sensitivity to lexical, syntactic, and compositional information in representing semantic information at the sentence level, while also indicating that details of how semantic features are extracted are critical for constructing brain-like sentence representations.

### Neuroscience of semantics

Our neuroimaging results show that linguistic information is represented across large parts of the cortex beyond the language network, including the default mode network that has been implicated in semantic processing in previous studies (Hamilton et al., 2011; Kuhnke et al., 2023; Fernandino and Binder, 2024). This supports previous studies which have found that processing of lexical semantics is intermingled with syntactic and structural processing (Shain et al., 2024; Blank and Fedorenko, 2020). One interesting supplementary finding is that the temporal regions of the language network tended to show somewhat larger effects (i.e. more negative Mean-CN correlations and higher VerbNet-CN correlations over the block diagonal pairs) compared to frontal regions of the network (see Figure 5). This aligns with several previous studies which have similarly found regions of the temporal lobe, especially the superior temporal sulcus, to play a prominent role in compositional and sentence processing (Fairhall, 2024; Wang et al., 2016; Brennan et al., 2016).

We also found a robust ‘minimum sentence length’ effect, whereby long sentences elicit very similar brain activity regardless of their lexical content or overall meaning (see Figure 3a and b). This effect is specific to long sentences and does not arise for pairs consisting of short or medium-length sentences. Though we are not aware of this result having been reported using RSA, previous studies using other methodologies have found that activation of the language network increases with sentence length (Shain et al., 2024; Nelson et al., 2017; Schuster et al., 2020; Woolnough et al., 2023). The cause of this effect remains unclear. It may partly be explained by visual similarity of long sentences; however, we observe no similar effect that might be expected for the visual similarity of short or medium sentences. Furthermore, the minimum length effect is also evident in many brain regions outside the visual cortex, including the language network and various frontal regions (see Figure 3). We speculate that the effect may be driven by multiple causes, including increased cognitive processing or memory load for processing longer sentences, greater depth of processing elicited by semantically richer stimuli (Deniz et al., 2023), or additional processing required for compositional combination of a larger number of sentence components. It is also possible that the structural similarity of longer sentences in our study, which all contain a similar set of semantic roles, results in similar brain representations even when the sentences do not have similar overall meanings. If so, this would indicate that extracting semantic features is important for brain processing of sentences even aside from lexical similarity. Further research will be required to disentangle the relative impacts of these distinct processes.

### Limitations

Our study has several limitations. First, we found a surprisingly low correlation between behavioural ratings and brain activations (see Figure 2). This may be partly explained by differences in task structure. In the behavioural experiment, participants viewed many pairs of related sentences and were explicitly asked to pay attention to differences in the words of each sentence. Conversely, in the fMRI task, participants (who were not the same as the behavioural task participants) read one sentence at a time without an explicit comparison. In addition, we suspect that presentation of so many sentence pairs with highly similar structures may have biased the way in which participants rated sentence similarity. Modifications to the behavioural task to mitigate these aspects may reduce the divergence between behavioural and brain findings.

Second, our stimulus set consists of a relatively small selection of sentences, which follow broadly similar structure. Our aim in this study was to disentangle the effects of lexical similarity from structural similarity in realistic sentences, and as such we did not attempt to compile a representative sample of sentences from natural dialogue. In future work, we hope to investigate the extent to which our results generalise to more complex and varied types of sentences.

Third, we analysed brain representations of sentence meaning over a single contiguous 3 s interval. This is a substantial simplification of sentence processing, which takes place dynamically over time as words are successively integrated to form progressively more complex and structured representations (Li et al., 2024; Shain et al., 2024; Baron et al., 2010; Frankland and Greene, 2020b; Desbordes et al., 2024). While our approach makes an important contribution and builds upon previous studies comparing syntactic parse trees with brain data (Nelson et al., 2017; Stanojević et al., 2023; Fresen et al., 2024), additional work is needed to link model representations to the dynamic cascade of brain activity during sentence processing.

### Conclusion

Our results provide important new insights about how sentence structure is represented in the brain. The simple Mean-CN model, which ignores sentence structure, was a very poor match to brain activity when evaluated against the block diagonal subset of sentences (the sentence pairs designed to be difficult for models which do not represent sentence structure). While transformers were a much better match to brain activity than the Mean-CN model, correlations were still negative, indicating that transformer representations were still a poor match to brain representations. In line with our preregistered prediction, we found that the VerbNet-CN model best matched brain representations, thereby providing evidence that the brain incorporates structured information from semantic roles when representing sentence meaning. Even though transformer models clearly do encode semantic role information, the fact that their hidden layers fail to show the similarity structure of human BOLD responses indicates that transformers do not represent this information in the same manner as the human brain. Our results therefore highlight the important lesson that human-like linguistic behaviour does not entail human-like linguistic representations.

## Methods

### Stimuli and computational models

#### Sentence stimuli

A set of 108 sentences was handcrafted specifically for this study. Our aim was to develop a set of sentence pairs which systematically tested different combinations of lexical similarity and overall semantic similarity. This allows for better model discrimination by ensuring that only models sensitive to sentence structure are able to accurately differentiate sentence meaning, reducing the confound of lexical similarity.

The process by which sentences were constructed is summarised in Table 1. All sentences consisted of a single clause written in the active voice describing a specific event. Pronouns, proper nouns, and subordinate clauses were excluded for simplicity and to limit sources of syntactic variation. Sentences were produced by constructing systematic variations of an initial ‘base’ sentence by altering elements such as the subject, verb, and object, or adding modifiers like adjectives, location, or time. In an effort to explore different combinations of lexical and overall sentence similarity, several different categories of altered sentences were constructed. A small number of ‘same’ sentences were constructed by adding a single adjective with only minimal effect on sentence meaning, for example ‘the equipment’ becomes ‘the new equipment’. ‘Modified’ sentences were constructed by adding two or three modifier elements such as location, manner, or time when the event occurred, under the hypothesis that adding these modifier terms would reduce lexical similarity but have only a small effect on overall sentence meaning. ‘Substituted’ sentences were designed to investigate the effect of altering key sentence elements, such as changing the subject, object, or verb of the sentence.

Critical to the study design was construction of ‘swapped’ sentences, in which one or more pairs of words interchanged roles in the sentence, thereby ensuring that lexical similarity is high while similarity in overall sentence meaning is low. For example, if in the initial sentence the subject is ‘the cameraman’, the direct object ‘the equipment’, and the indirect object ‘the director’, then in the interchanged sentence the subject is now ‘the director’, the direct object is ‘the cameraman’, and the indirect object is ‘the equipment’. As with the ‘base’ sentence, the swapped sentences were also systematically varied through substitutions and addition of modifiers. The aim of this procedure was to develop a set of sentence pairs with gradations of similarity while approximately controlling for lexical similarity. Differences in meaning in these sets of sentences are therefore mostly attributable to sentence structure and semantic roles, not simply use of different words. The complete set of stimuli is provided in Appendix 1.

Using the methods described above, six distinct subsets each consisting of 18 related sentences were developed. This resulted in 5778 pairwise comparisons across all sentences, of which 4860 were ‘different’ sentence pairs and 918 were block diagonal sentence pairs of primary interest in this study. The RSA design matrix for all 108 sentences is shown in Figure 1a.

In our study preregistration (see https://osf.io/jme7x), we predicted that over the block diagonal set of sentence pairs, the VerbNet-CN model would have a higher correlation with brain representations than the average over the five specified transformers, which in turn would have a higher correlation than the Mean-CN model. We did not make predictions for any other models.

#### Word embedding models

In this study we compared four different approaches for representing sentence meaning. The baseline for all comparisons was the Mean-CN model in which sentence embeddings are constructed by elementwise averaging of ConceptNet word embeddings (Speer et al., 2017). We also evaluated two alternative models for combining word embeddings into sentence embeddings. Multiplicative (Mult) embeddings were constructed by adding one to each element of the word embeddings (to avoid negative numbers), then performing elementwise multiplication of the ConceptNet word embeddings. Convolutional (Conv) embeddings were constructed by adding one to each element of the ConceptNet word embeddings, then iteratively performing circular discrete convolution of each word embedding with the convolution of all previous word embeddings. For all three models based on ConceptNet word embeddings, sentence embeddings were constructed after removing a list of stop words containing words with little semantic content such as pronouns, modal verbs, conjunctions, and common prepositions. As a comparison, we also evaluated GloVe word embeddings (Pennington et al., 2014), using simple averaging over all words in each sentence. Cosine similarity was used to compute the similarity of each pair of sentence embeddings.

#### Transformer models

We computed the representations for a range of transformer architectures, along with the older InferSent LSTM model for comparison, as summarised in Table 2. As per our preregistration, for the statistical analysis, we averaged the RSA correlation with brain representations over five different transformer architectures: ERNIE 2.0, AMRBart, SentBERT, DefSent, and OpenAI. For all transformers, sentence embeddings were normalised by subtracting the mean and dividing by the standard deviation of each feature. This is motivated by research indicating that without normalisation, transformers tend to learn very anisotropic embeddings with a few dimensions dominating over all the others (Timkey and van Schijndel, 2021; Cai et al., 2021). Sentence similarities were computed using cosine similarity.

**Table 2.** Summary of models of sentence meaning analysed in this study.

Model name	Explanation	Citation
InferSent	A bi-directional LSTM trained on a variety of natural language inference tasks.	Conneau et al., 2017
USE	Universal Sentence Encodings, based on a transformer architecture trained on a range of language tasks. Sentence embedding constructed by averaging all token embeddings from the final layer.	Cer et al., 2018
ERNIE 2.0	A transformer based on the BERT architecture trained using multi-task learning. Sentence embedding constructed by averaging all token embeddings from the final layer.	Sun et al., 2020
SentBERT	A sentence transformer model with additional training to predict paired sentences from a large dataset. We used the mpnet embeddings.	Reimers and Gurevych, 2019
DefSent-cls	A RoBERTa-large transformer adapted to produce sentence embeddings with fine-tuning on about 100,000 words paired with their dictionary definitions.	Tsukagoshi et al., 2021
AMRBart	A sentence transformer architecture trained to encode AMR graphs.	Bai et al., 2022
S3BERT	A sentence transformer based on SentBERT with extra training to use AMR graph-based metrics to construct an overall similarity score.	Opitz and Frank, 2022b
OpenAI	Embeddings provided from the OpenAI API model text-embedding-ada-002, based on a large transformer with additional fine-tuning from human feedback.	Neelakantan et al., 2022
Llama 3	A decoder-only transformer architecture trained using multi-task learning with additional fine-tuning from human feedback. Embeddings extracted from the 8B model. Sentence embedding constructed by averaging all token embeddings from the final layer.	Grattafiori et al., 2024

#### Graph models

We adopted AMR as the primary graph-based approach for representing sentence meaning. We used the SapienzaNLP (Spring) AMR parser (Bevilacqua et al., 2021) to parse all sentences, as it is among the best-performing AMR parsers with freely available and easily implementable code. Evaluating syntax-based models using STS datasets requires a method for computing the similarity between the graphs for each sentence. While various techniques have been developed for converting graphs into vector embeddings, these have typically focused on knowledge databanks rather than natural language (Goyal and Ferrara, 2018; Rossi et al., 2021). Furthermore, we are interested in testing graph-based models of representing sentences more directly, rather than the embeddings produced from these graphs. As such, we analyse the similarity of AMR graphs using two existing methods for comparing graph similarity directly: SMATCH (Cai and Knight, 2013) and WWLK (Opitz et al., 2021), yielding the AMR-Smatch and AMR-WLK models, respectively. The SMATCH metric computes the number of matching triples (sets of three connected nodes) that two AMR graphs share in common relative to the total number of triples across both graphs. The WWLK metric uses a very different approach, first constructing a vector embedding for each node based on its connections to other nodes, then concatenating across all nodes in the graph, and finally computing the transformation distance between these two concatenated node vectors. In the main manuscript, we report the results for the more widely used SMATCH metric, as it achieved much higher correlations than the WWLK metric.

As a supplementary analysis, we also evaluated constituency parses produced using two different methods. In the first, we constructed a simple CFG to produce candidate parses of all sentences, with the most plausible parse manually selected from these candidates. In the second approach, we used the Berkeley Neural Parser as implemented in the Benepar Python library to automatically parse all sentences (Kitaev and Klein, 2018; Kitaev and Klein, 2021). To compare the similarity of these graphs, we used both the edit distance and subtree similarity metrics (Collins and Duffy, 2001).

#### Hybrid models

To compute representations for the VerbNet-CN hybrid model, we used the GPT-4 model of the OpenAI Chat Completions API to parse each of the 108 sentences by assigning parts of the sentence to one of eight semantic roles: Verb, Agent, Patient, Theme, Time, Manner, Location, Trajectory. After parsing by semantic role, we then computed embeddings for each semantic role as before, by averaging the static ConceptNet embeddings of each constituent word after the removal of stop words. Words that are not associated with any semantic role were discarded. As before, the result is a set of role embeddings, which constitutes the representation of the meaning of the sentence in terms of vector representations of each major semantic role.

To compute the similarity between two sentences, we first aligned the two sentences based on the semantic roles. Matching semantic roles were then accorded a similarity of 0.5 plus the computed cosine similarity between the rolewise embeddings. In cases where the semantic role was present in one sentence but not the other, a rolewise similarity of zero was used. Overall sentence similarity was computed as the weighted average of these eight rolewise similarities. We used fixed weights of 3 for the Verb, and 2 for Agent, Patient, and Theme, and 0.5 for Time, Manner, Location, and Trajectory, adopted from our previous study (Fodor et al., 2025).

To compute representations for the AMR-CN hybrid model (Fodor et al., 2025), we first parsed sentences using the SapienzaNLP (Spring) AMR parser (Bevilacqua et al., 2021). Each token in the sentence was then assigned an ‘AMR role’ in accordance with its location in the parse tree by concatenating all nested parse labels. Role similarities were computed as the cosine similarity between the averaged ConceptNet word embeddings for all tokens with the same AMR role in each sentence of a sentence pair. Finally, the overall sentence similarity was computed as average role similarity over all roles found.

### fMRI data collection

#### Participants

Thirty-nine participants (23 women, 14 men, 2 other) between the ages of 18 and 40 (mean = 22.2) were recruited from our university campus (The University of Melbourne) for the study. All self-identified as native speakers of English, and all but one (a last-minute replacement) identified as right-handed. Participants received $70 as compensation for their time, which corresponds to about $23 per hour for a 3 hr session. All participants provided informed consent in written form prior to collection of the fMRI data. Nine participants were excluded from the main analysis: seven for scoring below 70% on the attention task (see details below) and two for head motion exceeding 4 mm maximum framewise deviation averaged over eight runs, leaving data from 30 participants for subsequent analysis. Note that owing to somewhat poorer performance of participants compared to those in our pilot, we lowered the cutoff slightly from the 75% stated in the preregistration, which led to the inclusion of a single additional participant who scored 73%. In Appendix 1—figure 21, we show that accuracy on attention check questions had a strong association with model correlations in line with our expectations.

The study protocol was approved by the University of Melbourne Human Research Ethics Committee (Reference Number: 2023-28035-47583-3).

#### Experimental task

While undergoing scanning, participants were presented with a set of 108 sentences, each shown one at a time. They were instructed simply to read each sentence and think about its meaning. Sentence timing was varied with the length of the sentence to allow sufficient time for reading longer sentences while avoiding leaving time for participants to engage in mind-wandering after reading the shorter sentences. The time for each sentence was computed using a quadratic formula in the number of characters, with parameters chosen based on feedback from pilot participants. Presentation time ranged from 2 to 7 s, with an average of 4.29 s per sentence. The inter-stimulus interval was selected from a uniform random distribution between 2 and 7 s, with an average of 4.5 s. The order of sentences was randomised separately for each participant, with 54 sentences presented during each 508 s run. The entire set of 108 sentences was presented every two runs, such that upon completion of all eight runs participants had seen each sentence four times. For five participants, only six runs were included, either because the participant did not complete the full scan or due to excessive head motion on the remaining two runs.

#### Attention task

To check attention and task engagement, participants were presented with four questions randomly distributed throughout each of the eight runs (40 questions total). All questions were four-option multiple choice questions relating to the meaning of the immediately preceding sentence. Each question, along with its potential answers, was displayed on screen for 5 s. Participants selected the answer using one of the two-button boxes held in each hand.

#### Image acquisition

The fMRI data was acquired using a 7 Tesla Siemens MAGNETOM scanner at the Melbourne Brain Centre (Parkville, Victoria) with a 32-channel radio frequency coil. The BOLD signal was measured using a multiband echo-planar imaging (EPI) sequence (TR = 800 ms, TE = 22.2 ms, FA = 45°). We acquired 636 volumes on each of the eight runs, each with 84 interleaved slices (thickness = 1.6 mm, gap = 0 mm, FOV = 208 mm, matrix = 130 × 130, multi-band factor = 6, voxel size = 1.6×1.6×1.6 mm^3^). Cardiac and respiratory traces were also recorded.

#### Preprocessing

Preprocessing was performed using fMRIprep with default parameters (Esteban et al., 2019). First, the T1-weighted (T1w) structural image was skull-stripped and normalised to the MNI152NLin2009cAsym standard space. Second, each of the eight BOLD runs was slice-time corrected and the volumes were motion-corrected by registering them to the single-band reference (SBRef) for each run. Distortion correction was applied by mapping field coefficients onto the reference image. All BOLD runs were then co-registered to the T1w reference, and resampled into the standard 1.6 mm MNI152NLin2009cAsym space. Full details of this process are given in Appendix 1.

#### GLM model

To model the brain activity pattern resulting from each sentence, a general linear model (GLM) was fitted using a boxcar function for each separate sentence convolved with the canonical HRF. This approach yields beta coefficients for each voxel and each distinct sentence stimulus. GLMs were fitted using GLMsingle (Prince et al., 2022), a sophisticated software package able to automatically detect and remove sources of noise, and also fit an appropriate HRF for each voxel.

A constant stimulus duration of 3 s was used for all stimuli for two reasons. First, GLMsingle does not support variable stimuli lengths. Second, participants will not form a full mental representation of a sentence until they finish reading it, so it is appropriate to only include the final portion of the stimulus for longer sentences.

In our preregistration, we stated we would extract the representation over the final 3 s for each stimulus. However, during the course of the study it became clear from participant feedback that the time provided for reading longer sentences was more than necessary, particularly for repeated trials. As such, in the main manuscript we instead report results for the middle 3 s of each stimulus. For example, for a 7 s sentence representations are evaluated during the window 2–5 s. We show in Appendix 1—figure 22 that our results are similar when using the final 3 s but with lower absolute magnitudes, presumably because participants begin to disengage with the task at the end of longer sentence presentations.

Three regressors of no interest were included in the GLM. The first was the number of characters displayed to the participant at any given time, as a control for the optical size of the visual stimulus. The final two regressors specified the timing of button presses for question responses, with one regressor each for left-hand and right-hand presses.

Regressions were run for each subject using the default parameters. Beta coefficients for each presentation of all 108 sentences were then extracted from the final ‘TYPED_FITHRF_GLMDENOISE_RR’ output of GLMsingle and averaged over all four presentations of each sentence.

### Behavioural data collection

#### Participants

A total of 507 participants (267 male, 223 female, and 17 other; age range, 18–45; mean age ± SD, 29.80±6.0) were recruited using the Prolific platform (https://www.prolific.com/). Participants were paid £4.50 for completing the task, which took an average of 22.5 min, amounting to an hourly rate of £11.96. All participants were self-declared native English speakers in Australia or the United States.

The study protocol was approved by the University of Melbourne Human Research Ethics Committee (Reference Number: 2023-23559-36378-6).

#### Survey task

Each participant provided similarity judgements on a 7-point Likert scale (1–7) for 102 sentence pairs randomly selected from the pool of all 5778 sentence pairs. As our primary interest was in the block diagonal sentences, we over-sampled from these sentence pairs relative to the other sentence pairs. As such, each participant rated 42 block diagonal sentence pairs and 60 other sentence pairs.

Given the inherent vagueness of the similarity judgement task, previous studies have noted that lengthy instructions on how to make similarity judgements are often unclear or may bias participant responses (Abe et al., 2022; Abdalla et al., 2023). Because our goal was to elicit intuitive judgements without imposing any particular framework which might influence results, we did not provide participants with any special training or instructions about how to assign ratings. Participants were simply instructed to ‘consider both the similarity in meaning of the individual words contained in the sentences and the similarity of the overall idea or meaning expressed by the sentences’. The full instructions given to participants can be found in Appendix 1.

In addition to the sentence pairs derived from the 108 experimental sentences, participants were also presented an additional 10 sentence pairs that served as an attention check. These stimuli consisted of either pairs of identical sentences (high similarity) or one simple sentence paired with a grammatically correct but nonsensical sentence (low similarity).

#### Preprocessing

We excluded all participants who failed more than two of the ten attention check items, resulting in 486 of 502 participants being retained. This amounted to 49,572 judgements, providing an average of 22 ratings for each block diagonal sentence pair and 6 for each of the other sentence pairs. Similarity judgements were averaged over participants and normalised between 0 and 1.

#### GPT-4 ratings

As an additional comparison to human similarity judgements, we also collected similarity ratings using the API of the GPT-4 model (Achiam et al., 2023). We passed each distinct pair of 5778 sentences to GPT-4 one at a time, to avoid any spurious effects of recent context. The prompt we used is given below:

‘You will be presented with two sentences. Your task is to judge how similar is the meaning of the two sentences. You will make this judgement by choosing a rating from 0 (most dissimilar) to 1 (most similar) to two decimal places. In providing your rating, consider both the similarity in meaning of the individual words contained in the sentences, as well as the similarity of the overall idea or meaning expressed by the sentences. Provide a numerical rating only; do not explain your answers. Here are the sentences.’

### Representational similarity analysis

#### Voxel selection

Voxel selection was performed in two different ways. To provide an overall brain representation, we extracted all voxels within the cortical mask from the MNI152NLin2009cAsym template. To eliminate potential confound from visual regions, we also constructed a cortical mask excluding voxels in visual cortical regions V1–V4 from the cortical mask. In our preregistration we stated that we would remove any voxels having an absolute correlation with sentence length greater than 0.5. However during our analysis we found this to be infeasible given the large number of voxels sensitive to sentence length. We subsequently became aware that several previous studies have found similar length effects in the language network (Nelson et al., 2017; Schuster et al., 2020; Woolnough et al., 2023). As such, we instead directly remove the visual cortex regions V1–V4 from analysis. As an additional check, we also performed all analyses controlling for the minimum sentence length, with the results shown in Appendix 1—figure 4. In addition, we also analysed voxels within a language ROI mask. The mask contains 26,000 voxels found to be primarily sensitive to linguistic stimuli in a series of previous experiments involving contrasting sentence stimuli with pseudowords (Schrimpf et al., 2021).

To identify voxels sensitive to sentence stimuli, the stability score was computed for each voxel as the average correlation between its time series of activity on different presentations of the stimuli (Just et al., 2010). All voxels within the mask with stability scores above a threshold of 0.07 were selected for computing RSA matrices. We show in Appendix 1—figures 19 and 20 that alternative stability thresholds yield similar results, though with higher magnitudes when higher thresholds are used.

Masks for cortical ROIs were constructed using the Glasser atlas (Glasser et al., 2016). Parcel indices included in each region were as follows: Dorsolateral prefrontal cortex: 67, 68, 71, 73, 83, 84, 85, 86, 87; dorsomedial prefrontal cortex: 26, 43, 63, 69; precuneus: 15, 27, 29, 30, 31, 45, 121, 142; posterior cingulate: 14, 32, 33, 34, 35, 38, 161, 162; primary visual cortex: 1, 4, 5, 6; primary somatosensory cortex: 9, 51, 52, 53.

#### Computing RSA matrices

For fMRI data, RSA matrices were computed by first normalising GLMsingle beta coefficients by subtracting the mean and dividing by the standard deviation for each voxel. Cosine similarities were then computed between the voxel representations of each sentence (using only the subset of included voxels) for each distinct pair of sentences, yielding an RSA matrix for each participant.

RSA matrices for computational models were computed differently depending on the model in question. For all vector-based models (including Mean and Transformer), sentence embeddings were extracted for each sentence and then normalised by subtracting the mean and dividing by the standard deviation for each dimension. Pairwise sentence similarities were then computed using cosine similarity between the corresponding embeddings.

As an additional check, for the vector-based models, we also computed similarities using the DIEM, which is designed to adjust for the effects of differences in dimensionality between embedding models (Tessari et al., 2024).

For models not entirely based on vector representations (i.e. the constituency parsers, the AMR-based models, and VerbNet-ConceptNet), we compute pairwise similarities as specified in Methods, Stimuli and computational models.

#### Data-model RSA correlations

RSA matrices for brain representations were compared with those of the computational models by calculating for each participant the partial Spearman correlation controlling for the difference in sentence lengths, then averaging over participants. We use the pingouin 0.5.4 Python package, which utilises the inverse covariance matrix for computing partial Spearman correlations. This has been proven more reliable than the alternative regression residuals technique when a subset of variables are discrete (see discussion at https://github.com/raphaelvallat/pingouin/issues/147, van Vliet et al., 2024). This is especially relevant for the AMR-Smatch model, as the SMATCH metric outputs a discrete similarity score.

In addition to the simple average across participants, we implemented an alternative method adapted from several previous studies (Wang et al., 2018; Fernandino et al., 2022; Handjaras et al., 2016), in which a group-averaged RSA matrix was first constructed by averaging pairwise sentence similarities over participants and then the correlation computed between each model RSA and this group-averaged RSA matrix.

For the simple average method, confidence intervals and statistical testing were performed using simple two-sided t-tests computed over participants. For the group average method, confidence intervals were estimated by bootstrapping over participants, performed 100 times. In the preregistration, we planned to perform bootstrapping over stimuli as well as over participants; however, in retrospect, we judged this to be inappropriate since our sentences were not a random sampling from some corpus, but were specially constructed to provide specified semantic and syntactic variation. For both methods, the Bonferroni correction was used to adjust for three independent model comparisons (Mean to Transformer, Transformer to VerbNet-CN, and VerbNet-CN to AMR-Smatch), yielding a significance level of α=0.05/3=0.0167.

We also computed the correlation between human-rated similarities and the brain RSA similarities, though we did not perform a statistical test as we had no prior hypothesis about this correlation.

### Searchlight RSA

To visualise the location of the cortical regions responsible for encoding sentence information, we implemented RSA-searchlight (Nili et al., 2014). Using the mne-rsa package (see https://users.aalto.fi/~vanvlm1/mne-rsa/index.html), we performed an 8 mm searchlight analysis over all voxels within the cortical mask. Images were smoothed with 5 mm FWHM and thresholded at z=2 using threshold-free cluster enhancement correction for display.

## Figures and Tables

**Appendix 1—figure 1.**
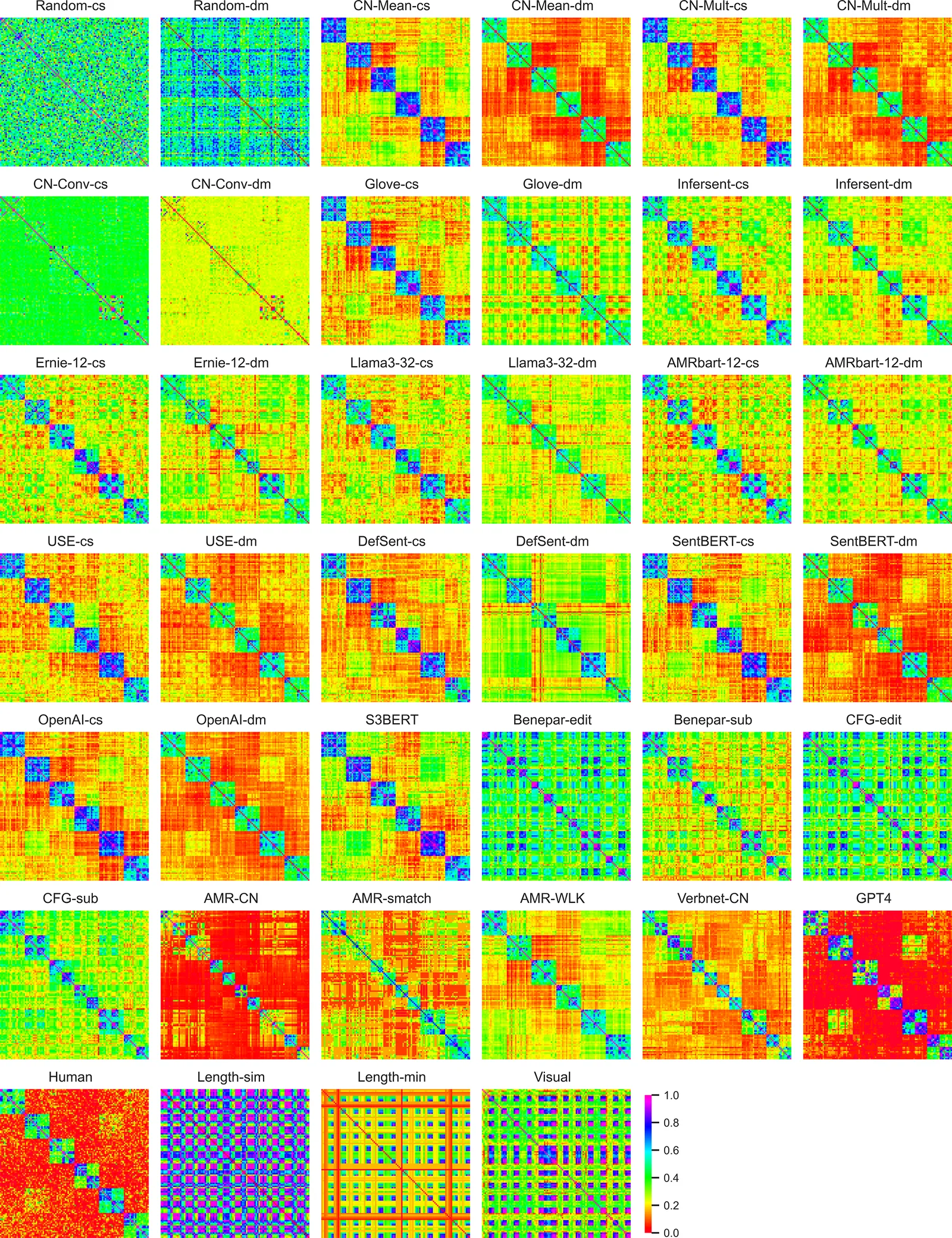
Representational similarity analysis (RSA) matrices for all computational models. Representational similarity matrices for all 21 models, as well as human and GPT-4 ratings, and three control variables related to sentence length and visual similarity. For vector-based models, two similarity metrics were used: cosine similarity (‘cs’) and dimension insensitive Euclidean metric (DIEM) similarity (‘dm’). Block diagonal patterns are visible for all models aside from random embeddings, indicating all models are sensitive to lexical similarity. Most transformers show only limited similarity to sentence structure, as evident from the lack of distinction between off-diagonal ‘swapped’ sentence pairs and on-diagonal ‘modified’ and ‘substituted’ sentence pairs. By contrast, the graph-based and the VerbNet-CN hybrid models generally show greater sensitivity to such structural differences.

**Appendix 1—figure 2.**
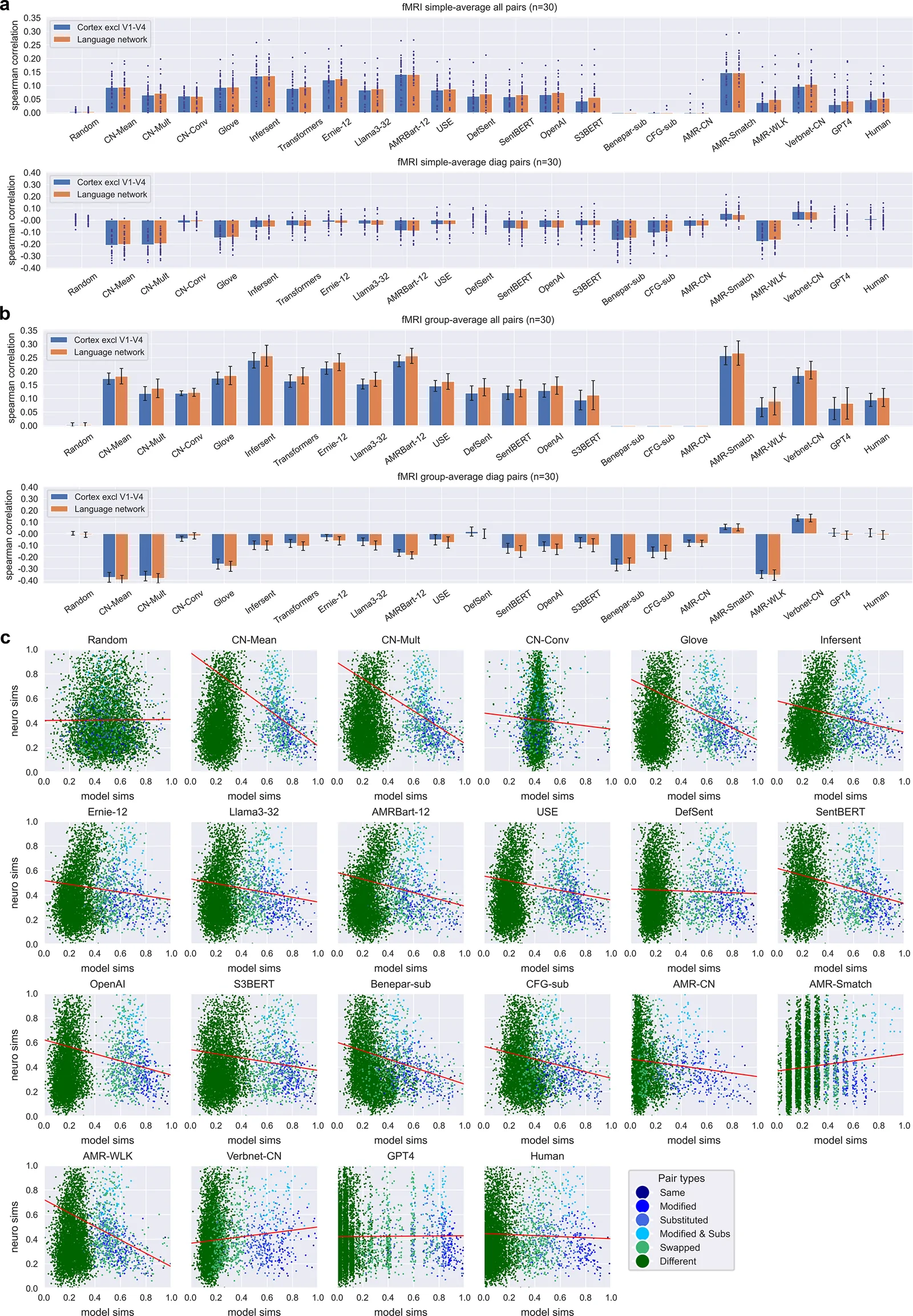
Correlations between all computational models and brain activity. Partial correlations between representational similarity analysis (RSA) matrices of all computational models and the brain RSA matrix, controlling for differences in sentence length. Blue bars show correlations computed over all stable (excluding visual regions V1–V4), while green bars show correlations for stable voxels in the language network. (**a**) Partial correlations for each individual participant shown as blue dots, with the simple average over individual correlations shown as a bar. (**b**) Partial correlations computed using the group-averaged RSA matrix. Error bars show 95% confidence intervals calculated by bootstrap resampling over participants. (**c**) Scatterplots showing the relationship between model similarities (horizontal axis) and group-average neural similarities (vertical axis) for all computational models. Each dot corresponds to a single pairwise similarity, scaled to between 0 and 1 for visualisation. While all sentence pairs are shown, regression lines (red) are computed over the block diagonal pairs only.

**Appendix 1—figure 3.**
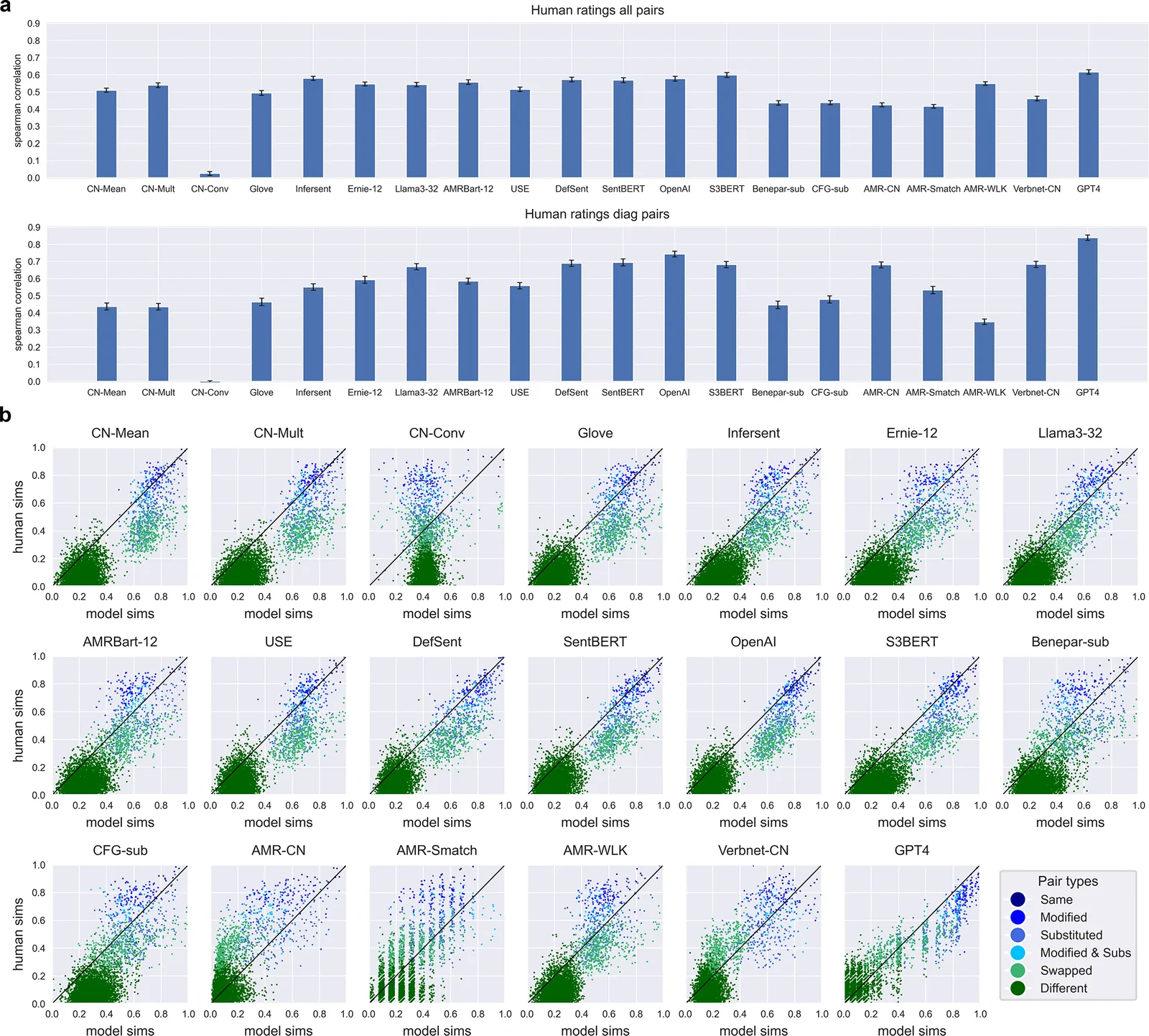
Correlations between behavioural ratings of sentence similarity and model similarities for all computational models. (**a**) Correlations between representational similarity analysis (RSA) matrices of all computational models and human-rated similarities computed over all sentence pairs (top) and block diagonal sentence pairs (bottom). Error bars show 95% confidence intervals calculated by bootstrap resampling over raters. (**b**) Scatterplots showing the relationship between model similarities (horizontal axis) and human rated similarities (vertical axis) for all computational models. Each dot corresponds to a single pairwise similarity. The 45-degree line (black) shows a hypothetical line of perfect fit between model and human similarities.

**Appendix 1—figure 4.**
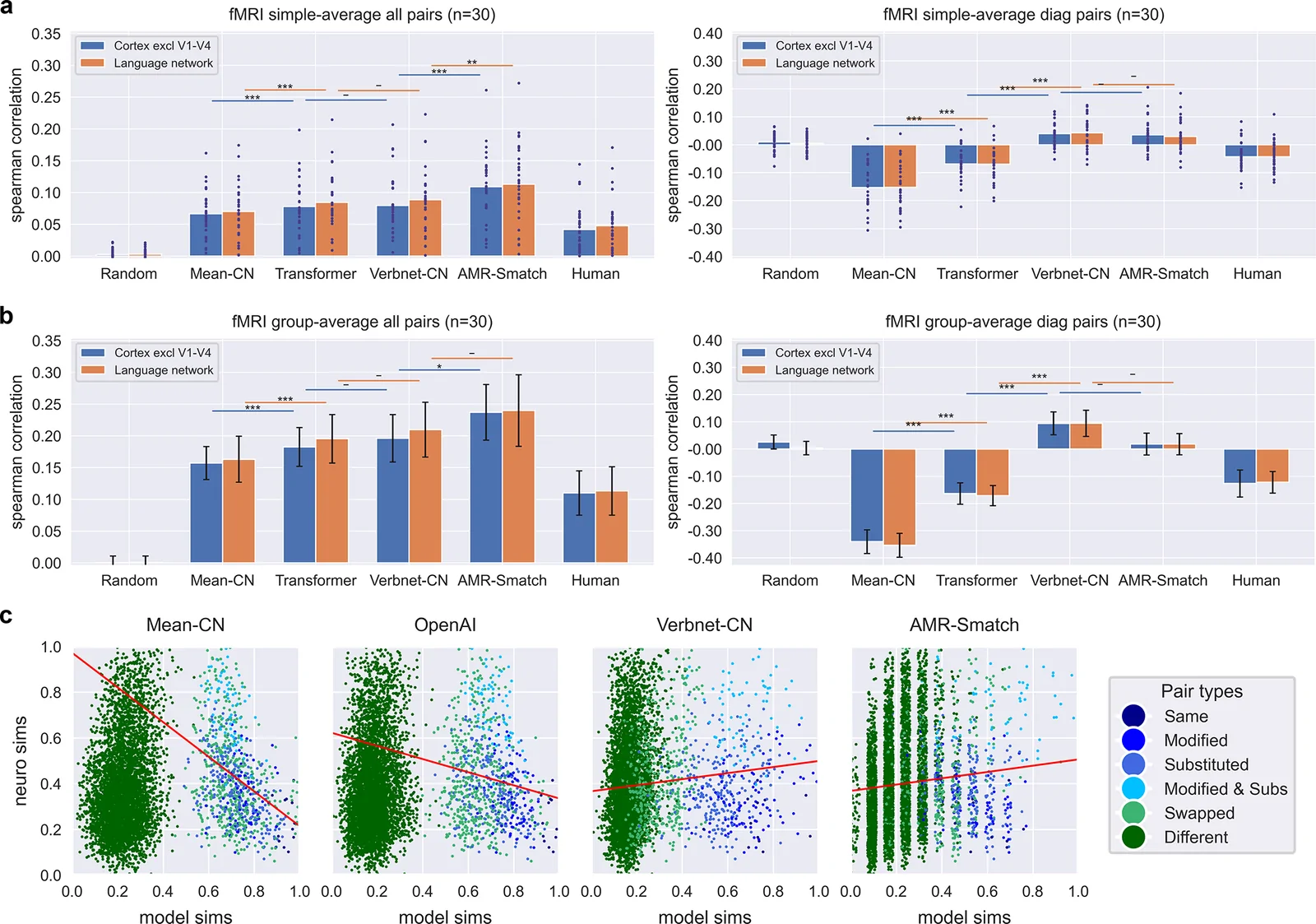
Model correlations with brain activity for all sentence pairs and the block diagonal subset of sentence pairs, with an additional control for minimum sentence length. Partial correlations between representational similarity analysis (RSA) matrices of five computational models and the brain RSA matrix, controlling for differences in sentence length and minimum sentence length. ‘Human’ refers to behavioural ratings. Blue bars show correlations computed over all stable (excluding visual regions V1–V4), while green bars show correlations for stable voxels in the language network. Notation for statistical significance: * for p<0.05, ** for p<0.01, and *** for p<0.001, with Bonferroni correction for three independent comparisons. (**a**) Partial correlations for each individual participant shown as blue dots, with the simple average over individual correlations shown as a bar. (**b**) Partial correlations computed using the group-averaged RSA matrix. Error bars show 95% confidence intervals calculated by bootstrap resampling over participants. (**c**) Scatterplots showing the relationship between model similarities (horizontal axis) and group-average neural similarities (vertical axis) for all four computational models. Each dot corresponds to a single pairwise similarity after regressing out the effects of sentence length similarity and minimum sentence length. Note that the displayed correlation for the AMR-Smatch is inaccurate owing to the discrete nature of SMATCH similarities, which leads to inconsistencies when using regression residuals to estimate brain similarities after controlling for length differences and the minimum sentence length. Similarities have been rescaled to between 0 and 1 for visualisation. While all sentence pairs are shown, regression lines (red) are computed over the block diagonal pairs only.

**Appendix 1—figure 5.**
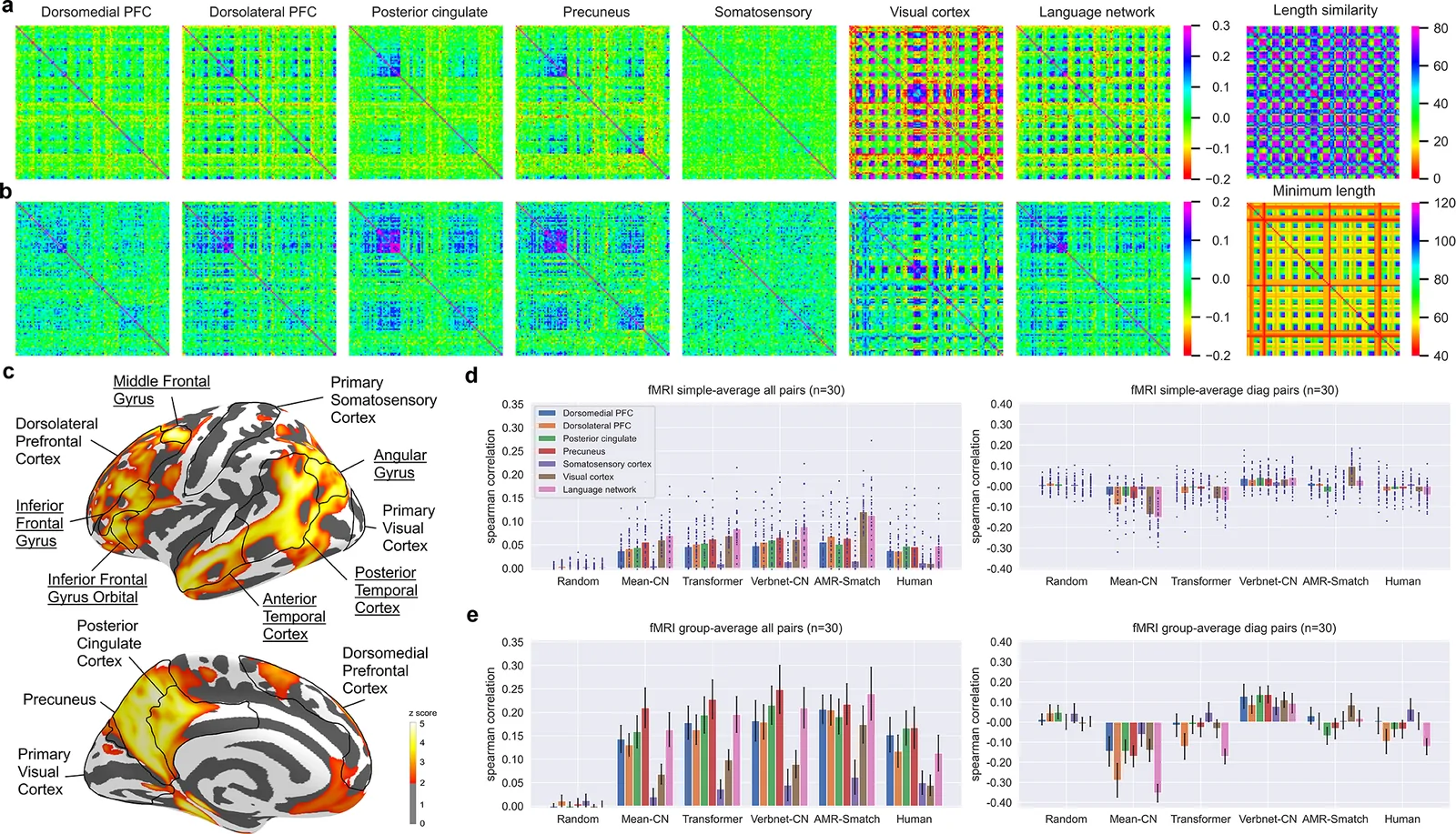
Comparison of sentence representations and model correlations across brain regions, with an additional control for minimum sentence length. (**a**) Representational similarity matrices for various cortical regions, computed controlling for differences in sentence length and minimum sentence length. (**b**) Representational similarity matrices for various cortical regions, computed controlling for difference in sentence length and minimum sentence length. Block diagonal structure is visible for all regions outside the visual cortex. (**c**) Searchlight representational similarity analysis (RSA) for hybrid model using 8 mm radius showing cortical regions of interest, with those part of the language network underlined. RSA correlations are thresholded at z=2. (**d**) Partial correlations controlling for differences in sentence length and minimum sentence length by cortical region, with the simple average over individual correlations shown as a bar. Error bars show 95% confidence intervals calculated by bootstrap resampling over participants. (**e**) Partial correlations controlling for differences in sentence length and minimum sentence length computed using the group-averaged RSA matrix, shown by cortical region. Error bars show 95% confidence intervals calculated by bootstrap resampling over participants.

**Appendix 1—figure 6.**
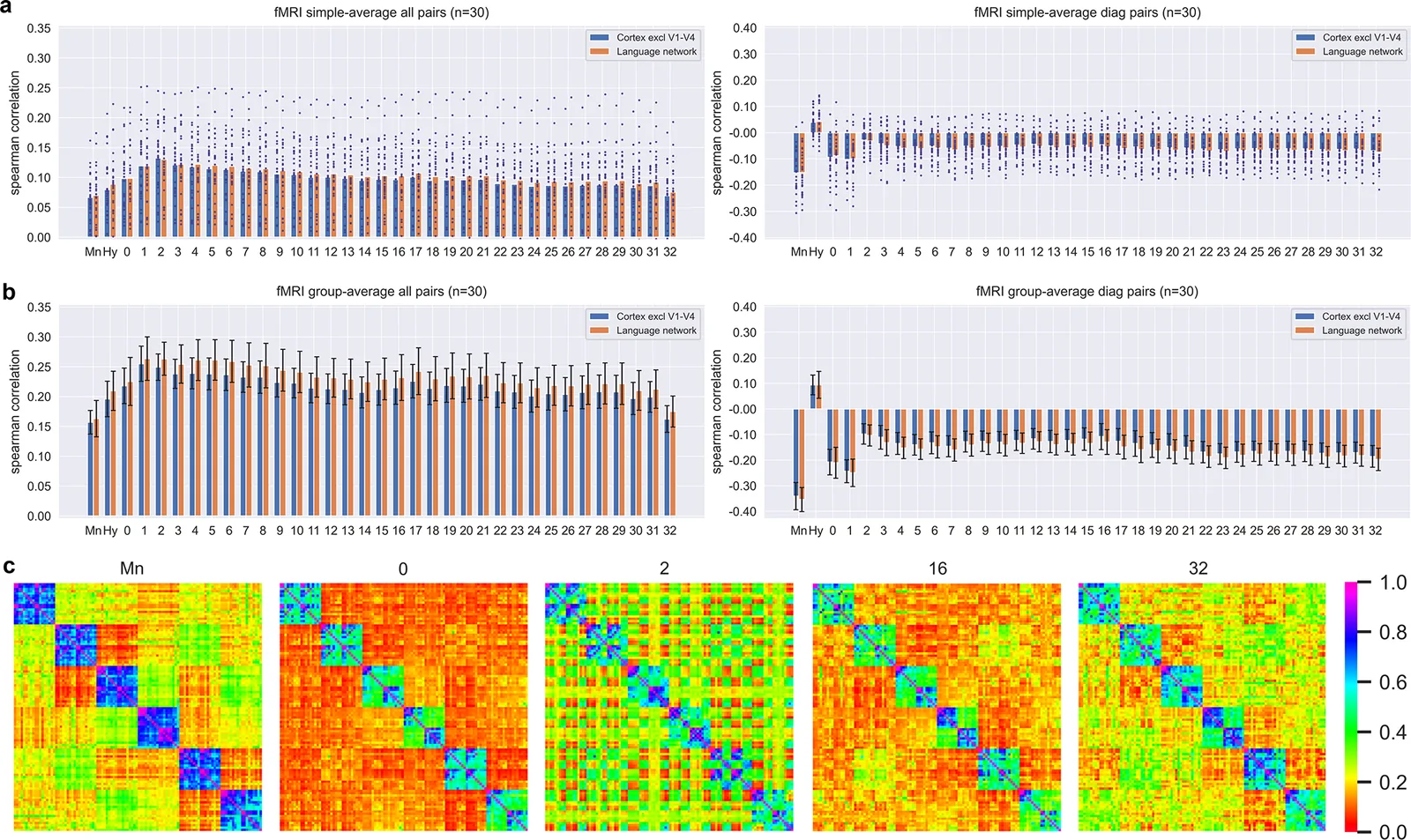
Correlations between individual fMRI data and Llama 3 layers, with an additional control for minimum sentence length. Average correlations between representational similarity analysis (RSA) matrices of each layer of Llama 3 and brain RSA matrix of each participant, controlling for the RSA similarity of visual cortex activity averaged over participants. Mean-CN (CN) and VerbNet-CN hybrid (VN) models are also shown for comparison. (**a**) Partial correlations for each individual participant shown as blue dots, with the simple average over individual correlations shown as a bar. (**b**) Partial correlations computed using the group-averaged RSA matrix. Error bars show 95% confidence intervals calculated by bootstrap resampling over participants. (**c**) RSA matrices for the Mean-CN and VerbNet-CN models, along with selected layers of Llama 3, computed with an additional control for minimum sentence length.

**Appendix 1—figure 7.**
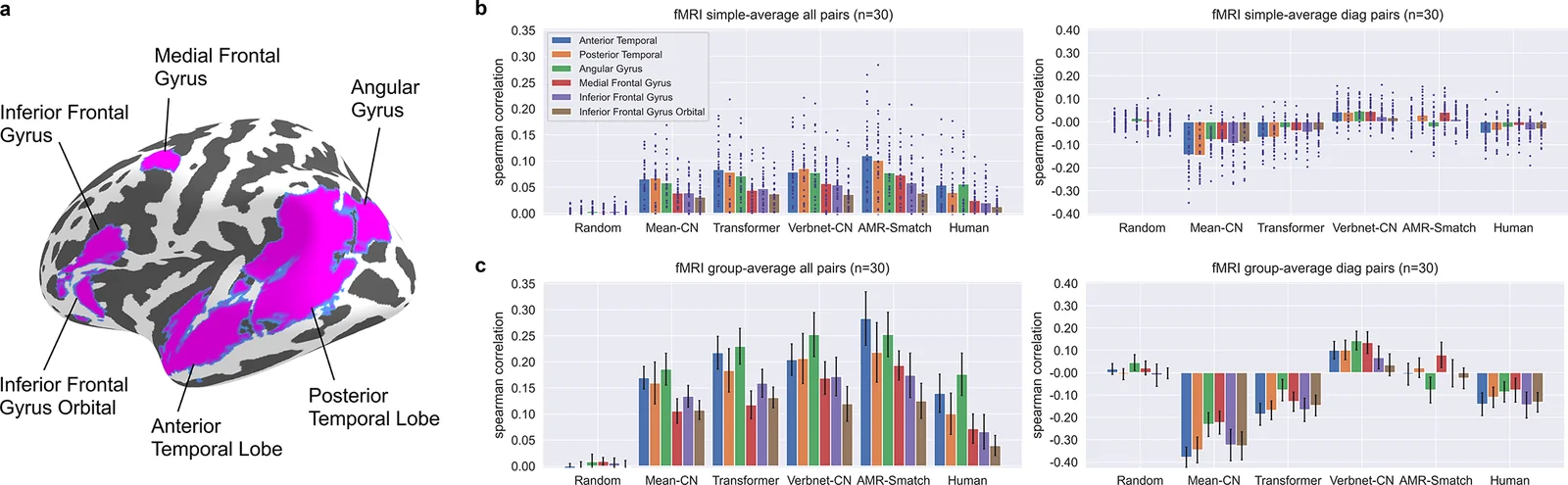
Comparison of model correlations across subregions of the language network, with an additional control for minimum sentence length. (**a**) Regions within the language network. (**b**) Partial correlations controlling for differences in sentence length and minimum sentence length shown by language network subregion, with each individual participant shown as blue dots, and the simple average over individual correlations shown as a bar. (**c**) Partial correlations controlling for differences in sentence length and minimum sentence length computed using the group-averaged representational similarity analysis (RSA) matrix, shown by language network subregion. Error bars show 95% confidence intervals calculated by bootstrap resampling over participants.

**Appendix 1—figure 8.**
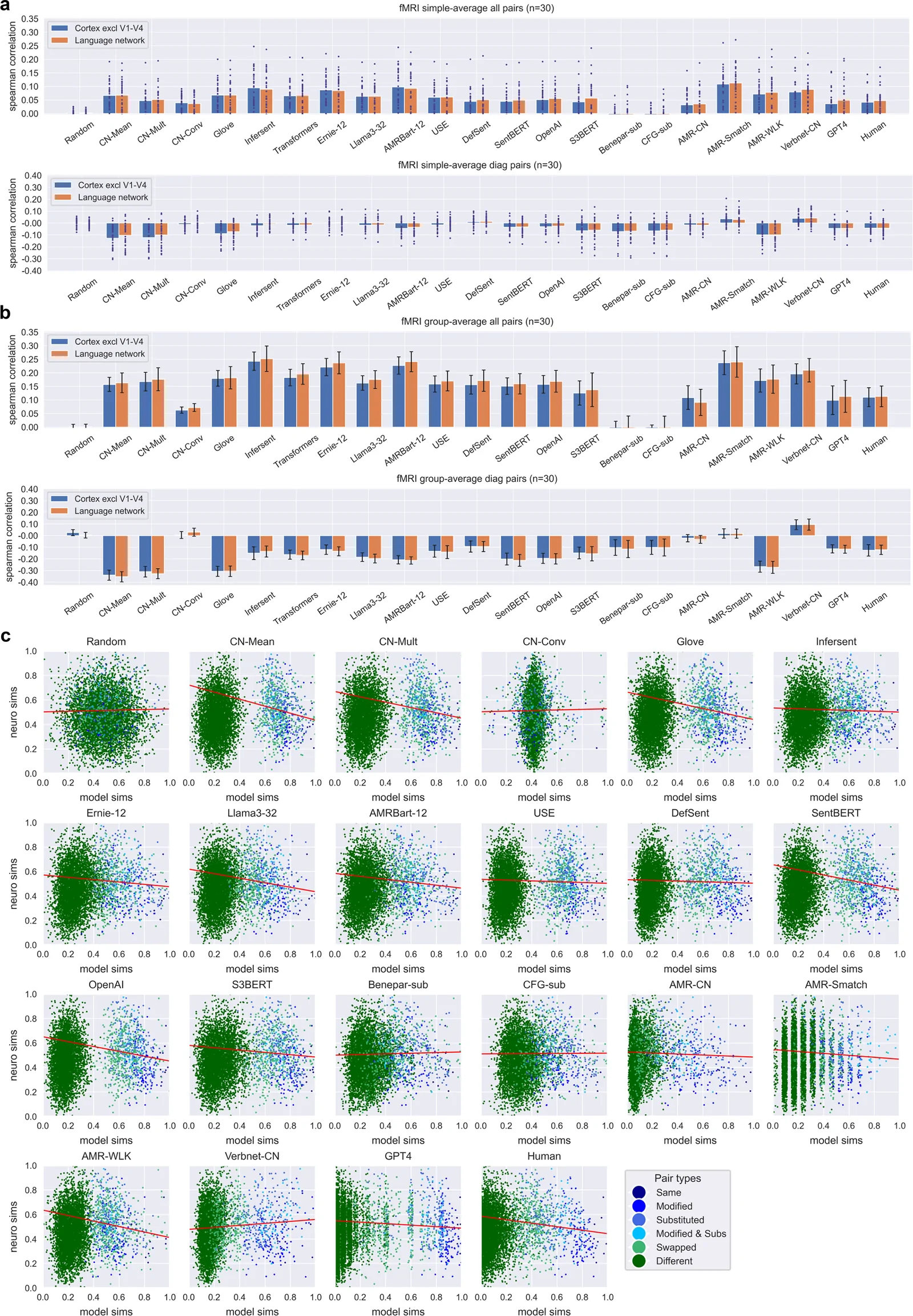
Correlations between all computational models and brain activity, with an additional control for minimum sentence length. Partial correlations between representational similarity analysis (RSA) matrices of all computational models and the brain RSA matrix, controlling for differences in sentence length and minimum sentence length. Blue bars show correlations computed over all stable (excluding visual regions V1–V4), while green bars show correlations for stable voxels in the language network. (**a**) Partial correlations for each individual participant shown as blue dots, with the simple average over individual correlations shown as a bar. (**b**) Partial correlations computed using the group-averaged RSA matrix. Error bars show 95% confidence intervals calculated by bootstrap resampling over participants. (**c**) Scatterplots showing the relationship between model similarities (horizontal axis) and group-average neural similarities (vertical axis) for all computational models. Each dot corresponds to a single pairwise similarity, scaled to between 0 and 1 for visualisation. While all sentence pairs are shown, regression lines (red) are computed over the block diagonal pairs only.

**Appendix 1—figure 9.**
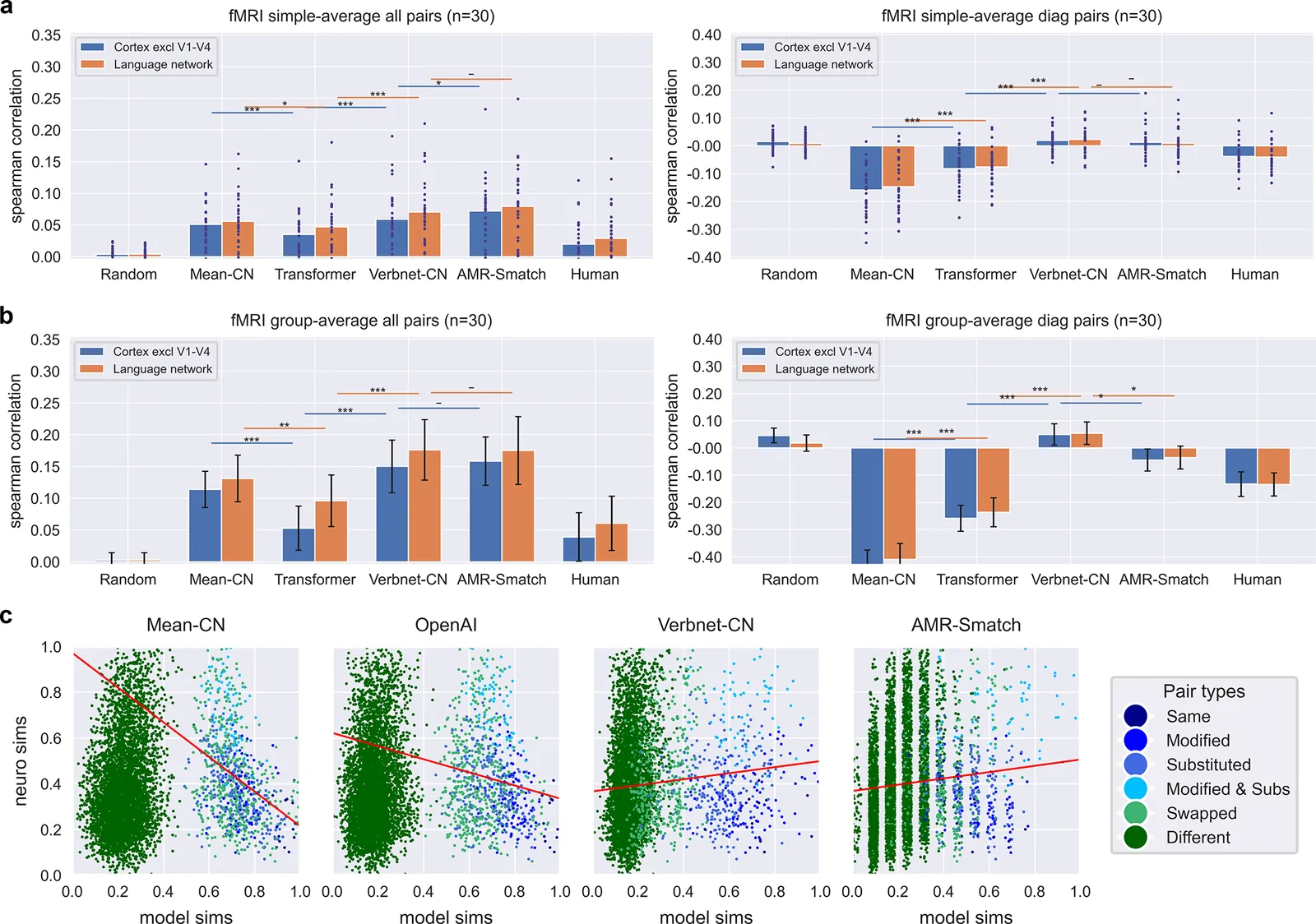
Model correlations with brain activity for all sentence pairs and the block diagonal subset of sentence pairs, with visual similarity control. Partial correlations between representational similarity analysis (RSA) matrices of five computational models and the brain RSA matrix, controlling for the RSA similarity of visual cortex activity averaged over participants. ‘Human’ refers to behavioural ratings. Blue bars show correlations computed over all stable (excluding visual regions V1–V4), while green bars show correlations for stable voxels in the language network. Notation for statistical significance: * for p<0.05, ** for p<0.01, and *** for p<0.001, with Bonferroni correction for three independent comparisons. (**a**) Partial correlations for each individual participant shown as blue dots, with the simple average over individual correlations shown as a bar. (**b**) Partial correlations computed using the group-averaged RSA matrix. Error bars show 95% confidence intervals calculated by bootstrap resampling over participants. (**c**) Scatterplots showing the relationship between model similarities (horizontal axis) and group-average neural similarities (vertical axis) for all four computational models. Each dot corresponds to a single pairwise similarity after regressing out the subject-averaged visual cortex similarities. Note that the displayed correlation for the AMR-Smatch is inaccurate owing to the discrete nature of SMATCH similarities, which leads to inconsistencies when using regression residuals to estimate brain similarities. Similarities have been rescaled to between 0 and 1 for visualisation. While all sentence pairs are shown, regression lines (red) are computed over the block diagonal pairs only.

**Appendix 1—figure 10.**
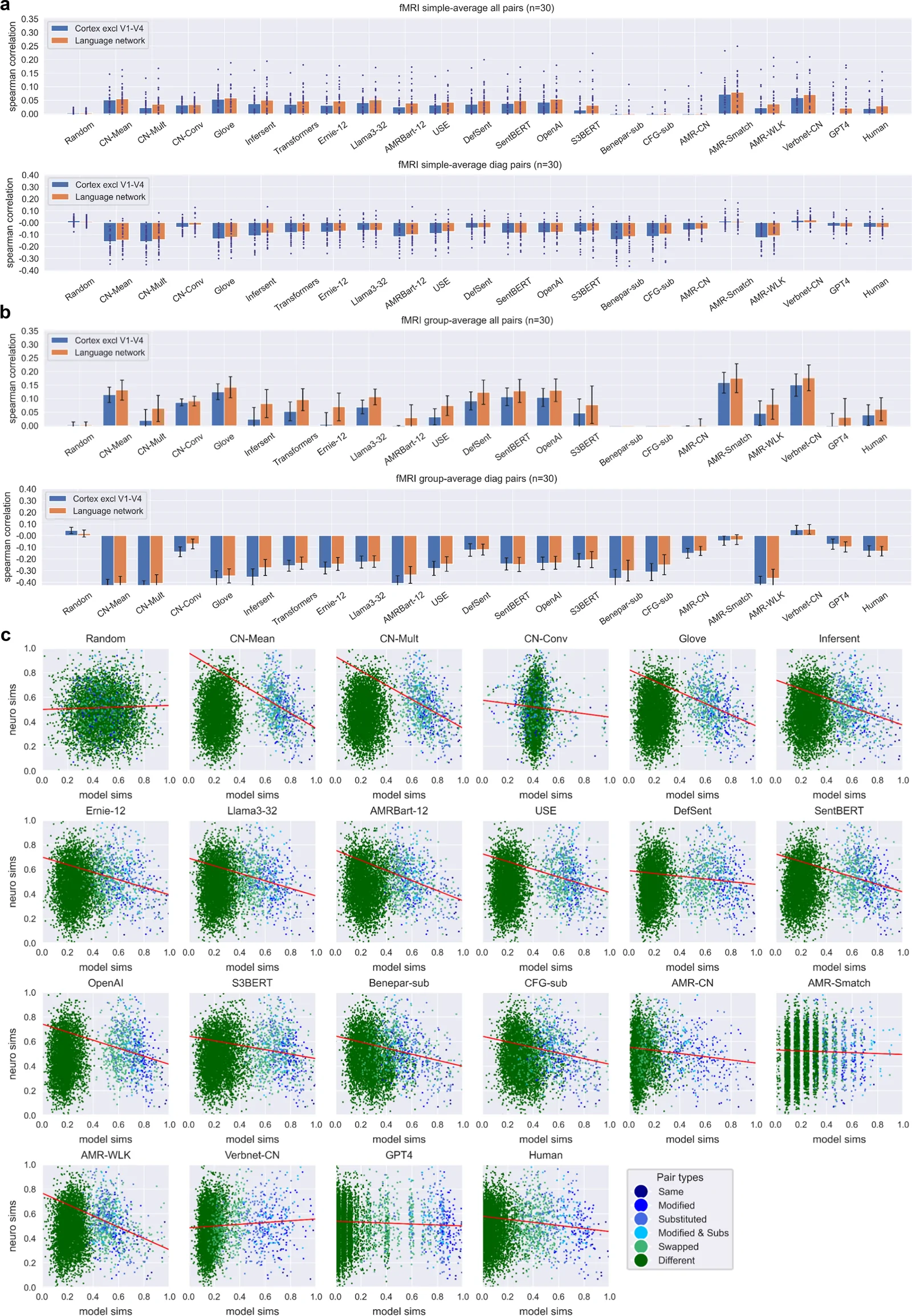
Correlations between all computational models and brain activity, with visual similarity control. Partial correlations between representational similarity analysis (RSA) matrices of all computational models and the brain RSA matrix, controlling for the RSA similarity of visual cortex activity averaged over participants. Blue bars show correlations computed over all stable (excluding visual regions V1–V4), while green bars show correlations for stable voxels in the language network. (**a**) Partial correlations for each individual participant shown as blue dots, with the simple average over individual correlations shown as a bar. (**b**) Partial correlations computed using the group-averaged RSA matrix. Error bars show 95% confidence intervals calculated by bootstrap resampling over participants. (**c**) Scatterplots showing the relationship between model similarities (horizontal axis) and group-average neural similarities (vertical axis) for all computational models. Each dot corresponds to a single pairwise similarity, scaled to between 0 and 1 for visualisation. While all sentence pairs are shown, regression lines (red) are computed over the block diagonal pairs only.

**Appendix 1—figure 11.**
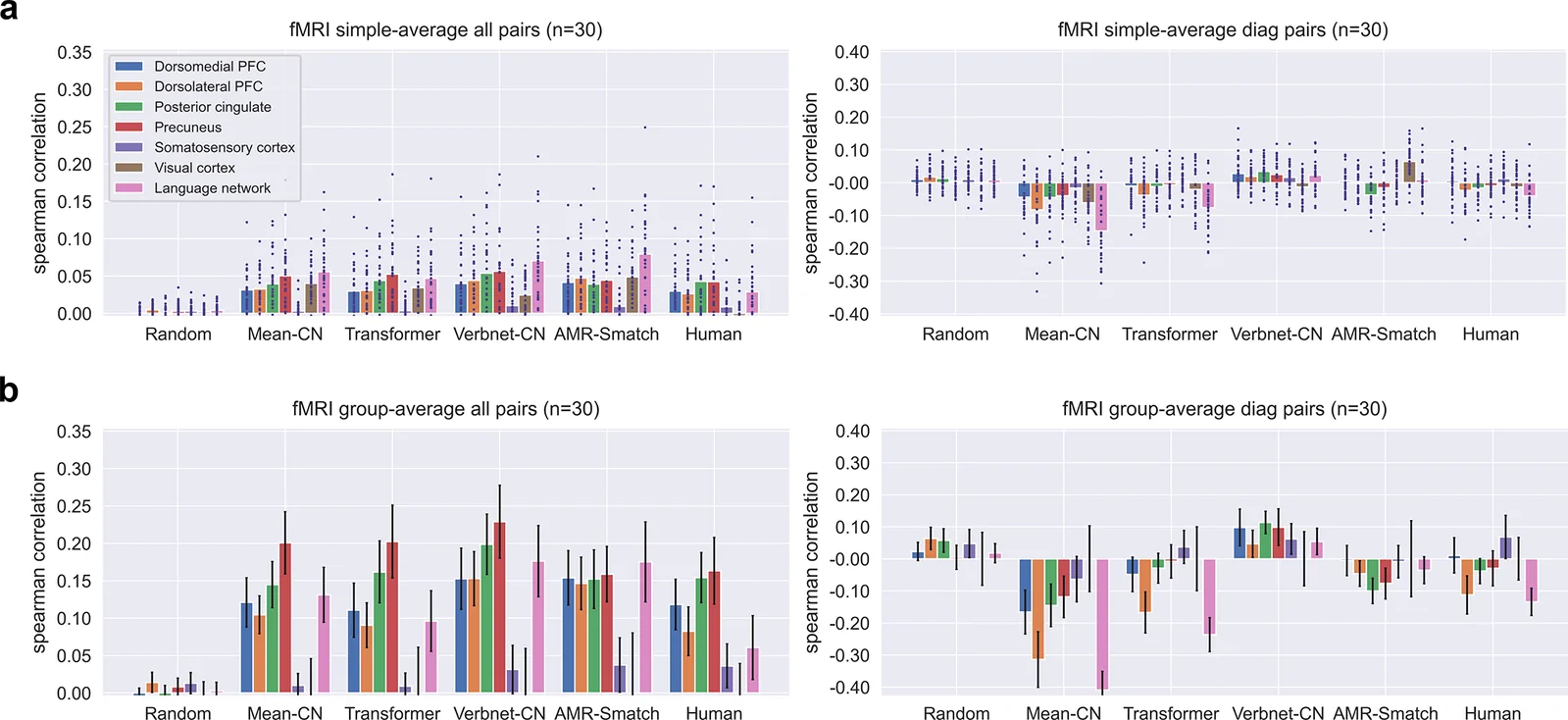
Comparison of sentence representations and model correlations across brain regions, with visual similarity control. (**a**) Partial correlations controlling for differences in sentence length and minimum sentence length by cortical region, with the simple average over individual correlations shown as a bar. Error bars show 95% confidence intervals calculated by bootstrap resampling over participants. (**b**) Partial correlations controlling for differences in sentence length and minimum sentence length computed using the group-averaged representational similarity analysis (RSA) matrix, shown by cortical region. Error bars show 95% confidence intervals calculated by bootstrap resampling over participants.

**Appendix 1—figure 12.**
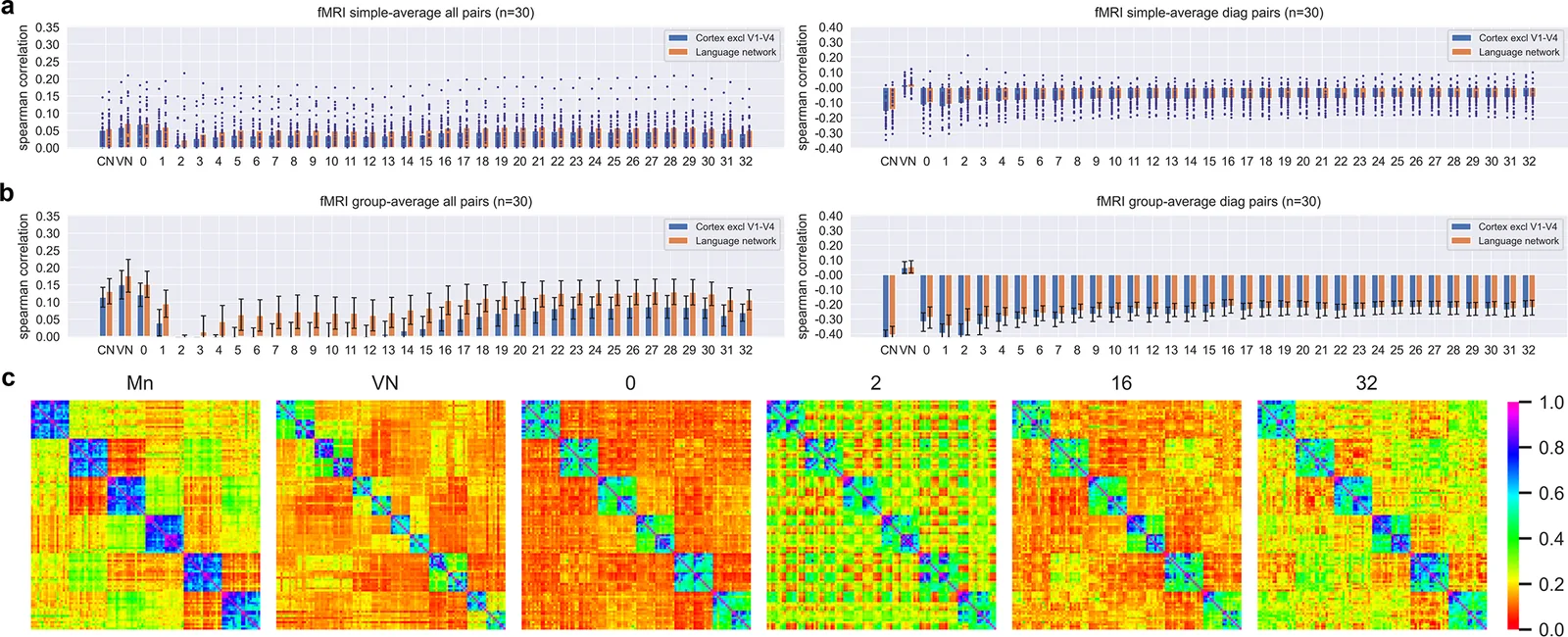
Correlations between individual fMRI data and Llama 3 layers, with visual similarity control. Average correlations between representational similarity analysis (RSA) matrices of each layer of Llama 3 and brain RSA matrix of each participant, controlling for the RSA similarity of visual cortex activity averaged over participants. Mean-CN (CN) and VerbNet-CN hybrid (VN) models are also shown for comparison. (**a**) Partial correlations for each individual participant shown as blue dots, with the simple average over individual correlations shown as a bar. (**b**) Partial correlations computed using the group-averaged RSA matrix. Error bars show 95% confidence intervals calculated by bootstrap resampling over participants. (**c**) RSA matrices for the Mean-CN and VerbNet-CN models, along with selected layers of Llama 3, computed controlling for visual similarity.

**Appendix 1—figure 13.**
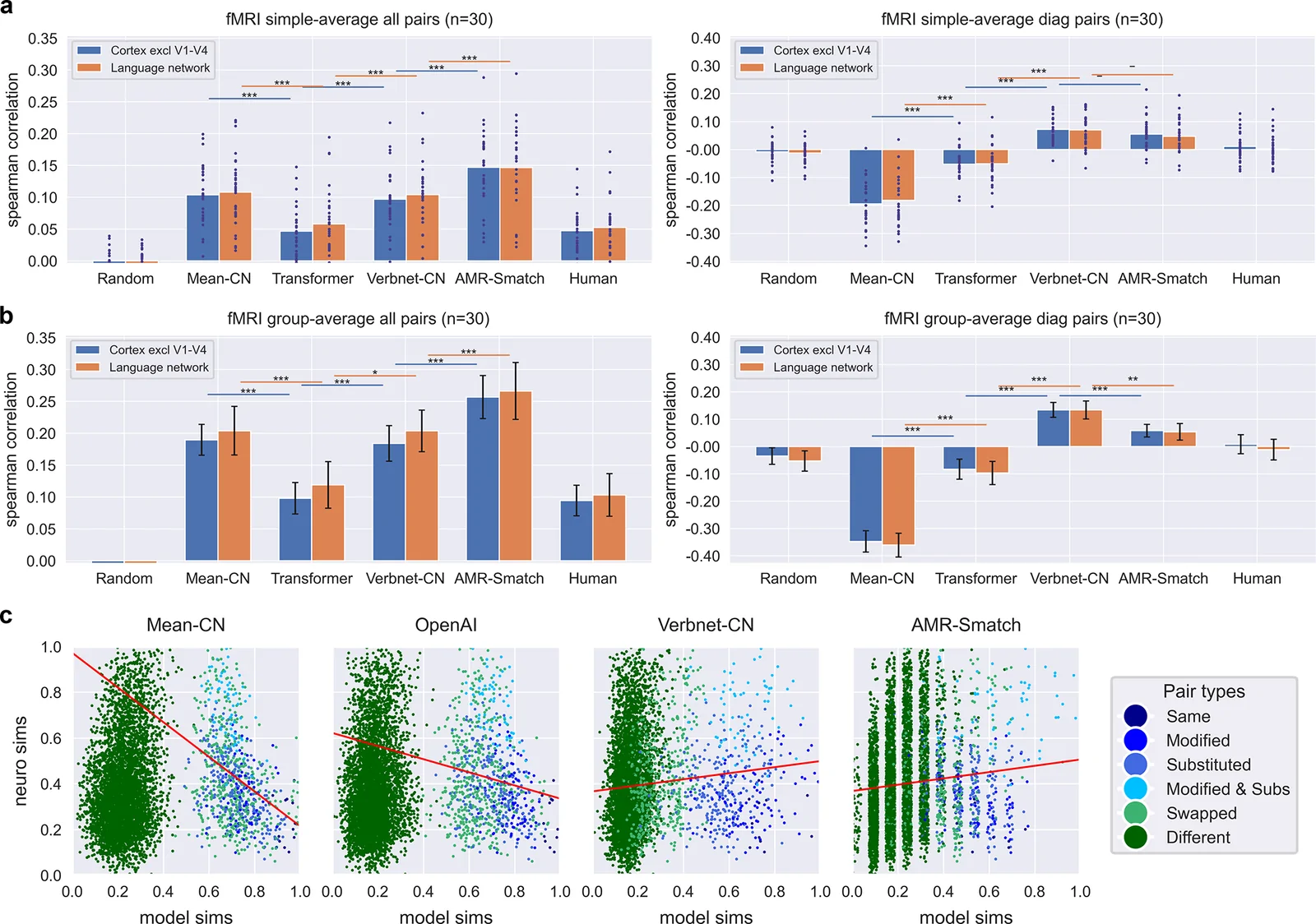
Model correlations with brain activity for all sentence pairs and the block diagonal subset of sentence pairs using the dimension insensitive Euclidean metric (DIEM) similarity metric. Partial correlations between representational similarity analysis (RSA) matrices of five computational models and the brain RSA matrix, controlling for differences in sentence length. ‘Human’ refers to behavioural ratings. Blue bars show correlations computed over all stable (excluding visual regions V1–V4), while green bars show correlations for stable voxels in the language network. Notation for statistical significance: * for p<0.05, ** for p<0.01, and *** for p<0.001, with Bonferroni correction for three independent comparisons. (**a**) Partial correlations for each individual participant shown as blue dots, with the simple average over individual correlations shown as a bar. (**b**) Partial correlations computed using the group-averaged RSA matrix. Error bars show 95% confidence intervals calculated by bootstrap resampling over participants. (**c**) Scatterplots showing the relationship between model similarities (horizontal axis) and group-average neural similarities (vertical axis) for all four computational models. Each dot corresponds to a single pairwise similarity after regressing out the effects of sentence length similarity and minimum sentence length. Note that the displayed correlation for the AMR-Smatch is inaccurate owing to the discrete nature of SMATCH similarities, which leads to inconsistencies when using regression residuals to estimate brain similarities after controlling for length differences and the minimum sentence length. Similarities have been rescaled to between 0 and 1 for visualisation. While all sentence pairs are shown, regression lines (red) are computed over the block diagonal pairs only.

**Appendix 1—figure 14.**
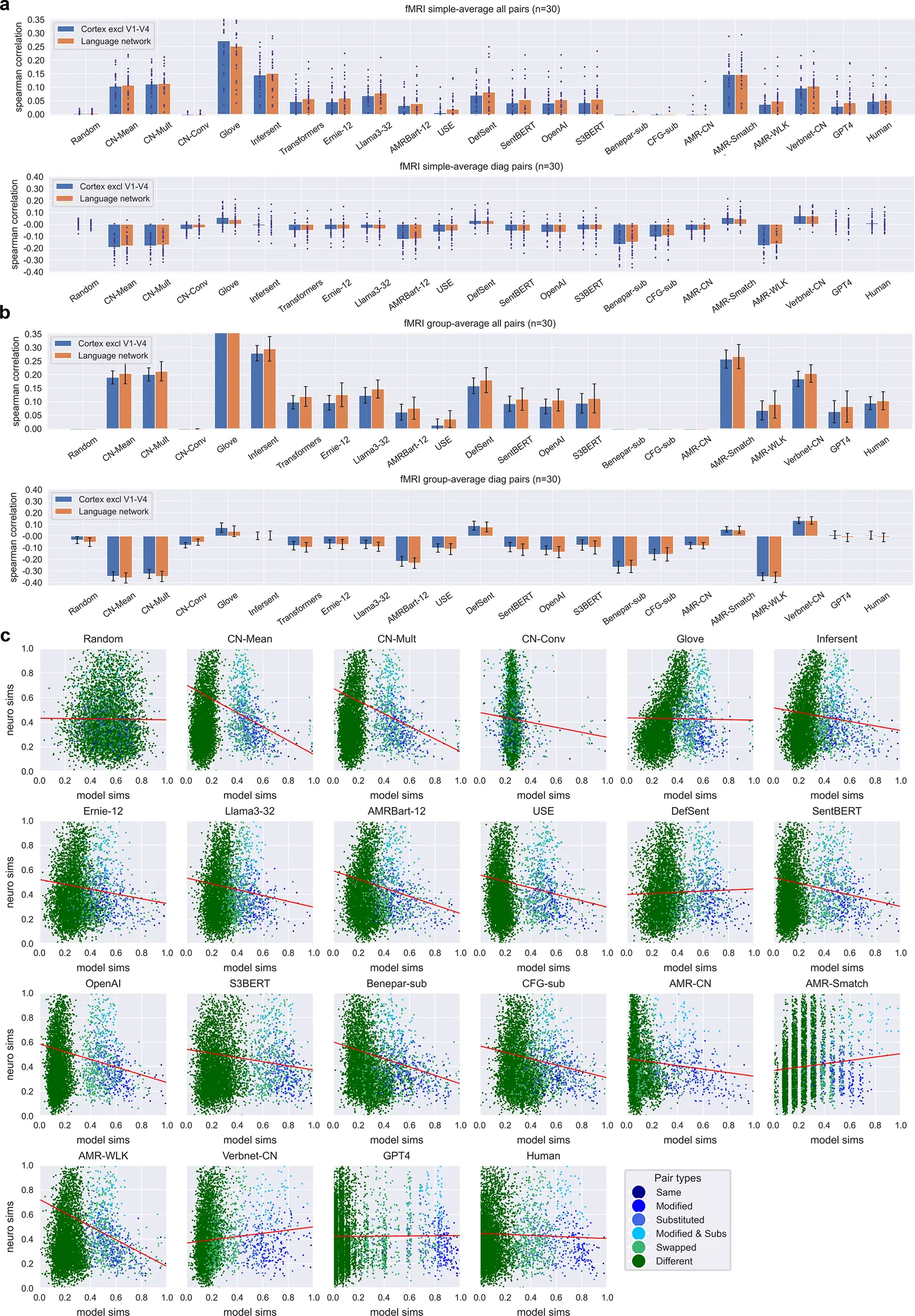
Correlations between all computational models and brain activity using dimension insensitive Euclidean metric (DIEM) similarity metric. Partial correlations between representational similarity analysis (RSA) matrices of all computational models and the brain RSA matrix, controlling for differences in sentence length. Blue bars show correlations computed over all stable (excluding visual regions V1–V4), while green bars show correlations for stable voxels in the language network. (**a**) Partial correlations for each individual participant shown as blue dots, with the simple average over individual correlations shown as a bar. (**b**) Partial correlations computed using the group-averaged RSA matrix. Error bars show 95% confidence intervals calculated by bootstrap resampling over participants. (**c**) Scatterplots showing the relationship between model similarities (horizontal axis) and group-average neural similarities (vertical axis) for all computational models. Each dot corresponds to a single pairwise similarity, scaled to between 0 and 1 for visualisation. While all sentence pairs are shown, regression lines (red) are computed over the block diagonal pairs only.

**Appendix 1—figure 15.**
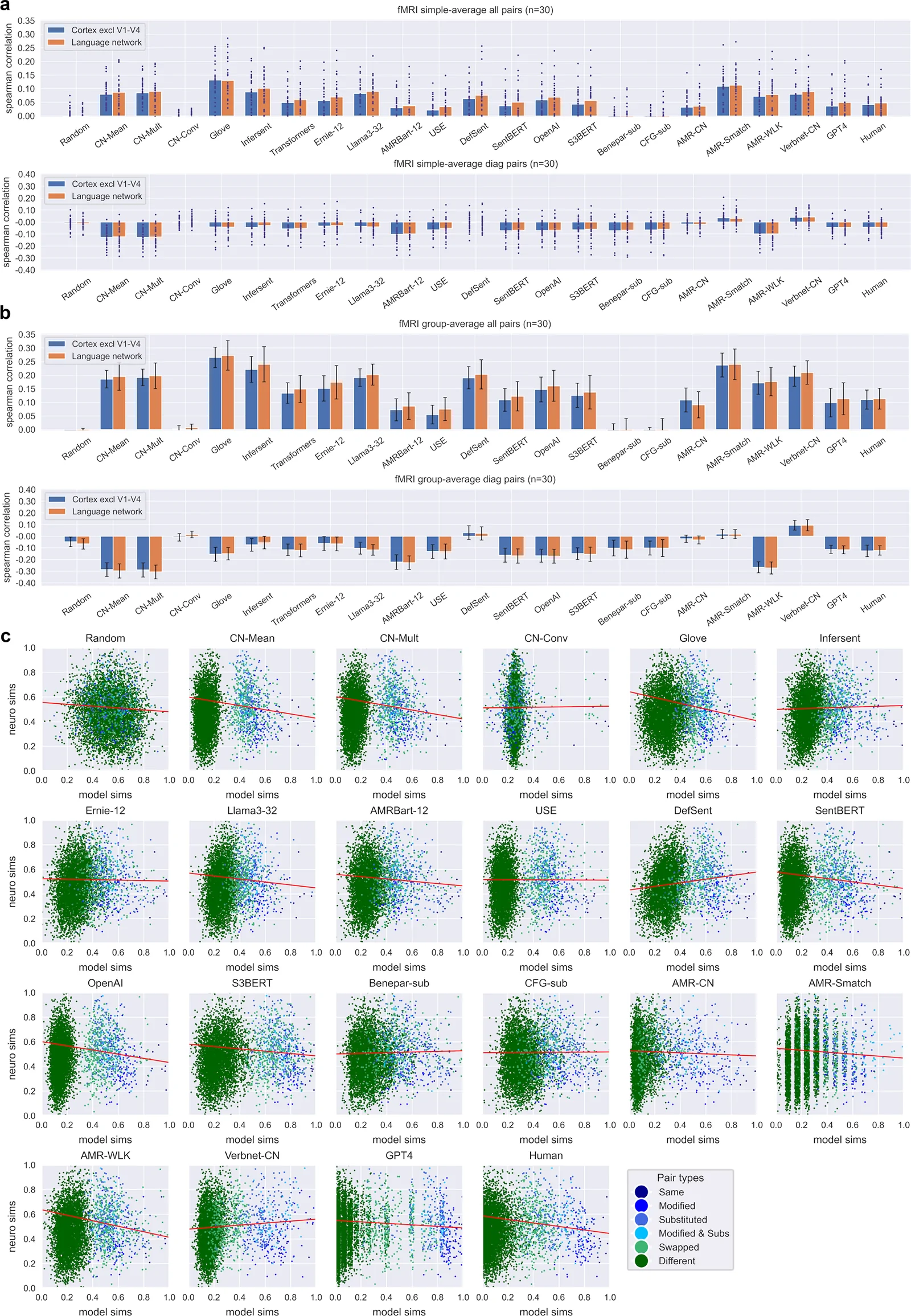
Correlations between all computational models and brain activity using dimension insensitive Euclidean metric (DIEM) similarity metric, with an additional control for minimum sentence length. Partial correlations between representational similarity analysis (RSA) matrices of all computational models and the brain RSA matrix, controlling for differences in sentence length and minimum sentence length. Blue bars show correlations computed over all stable (excluding visual regions V1–V4), while green bars show correlations for stable voxels in the language network. (**a**) Partial correlations for each individual participant shown as blue dots, with the simple average over individual correlations shown as a bar. (**b**) Partial correlations computed using the group-averaged RSA matrix. Error bars show 95% confidence intervals calculated by bootstrap resampling over participants. (**c**) Scatterplots showing the relationship between model similarities (horizontal axis) and group-average neural similarities (vertical axis) for all computational models. Each dot corresponds to a single pairwise similarity, scaled to between 0 and 1 for visualisation. While all sentence pairs are shown, regression lines (red) are computed over the block diagonal pairs only.

**Appendix 1—figure 16.**
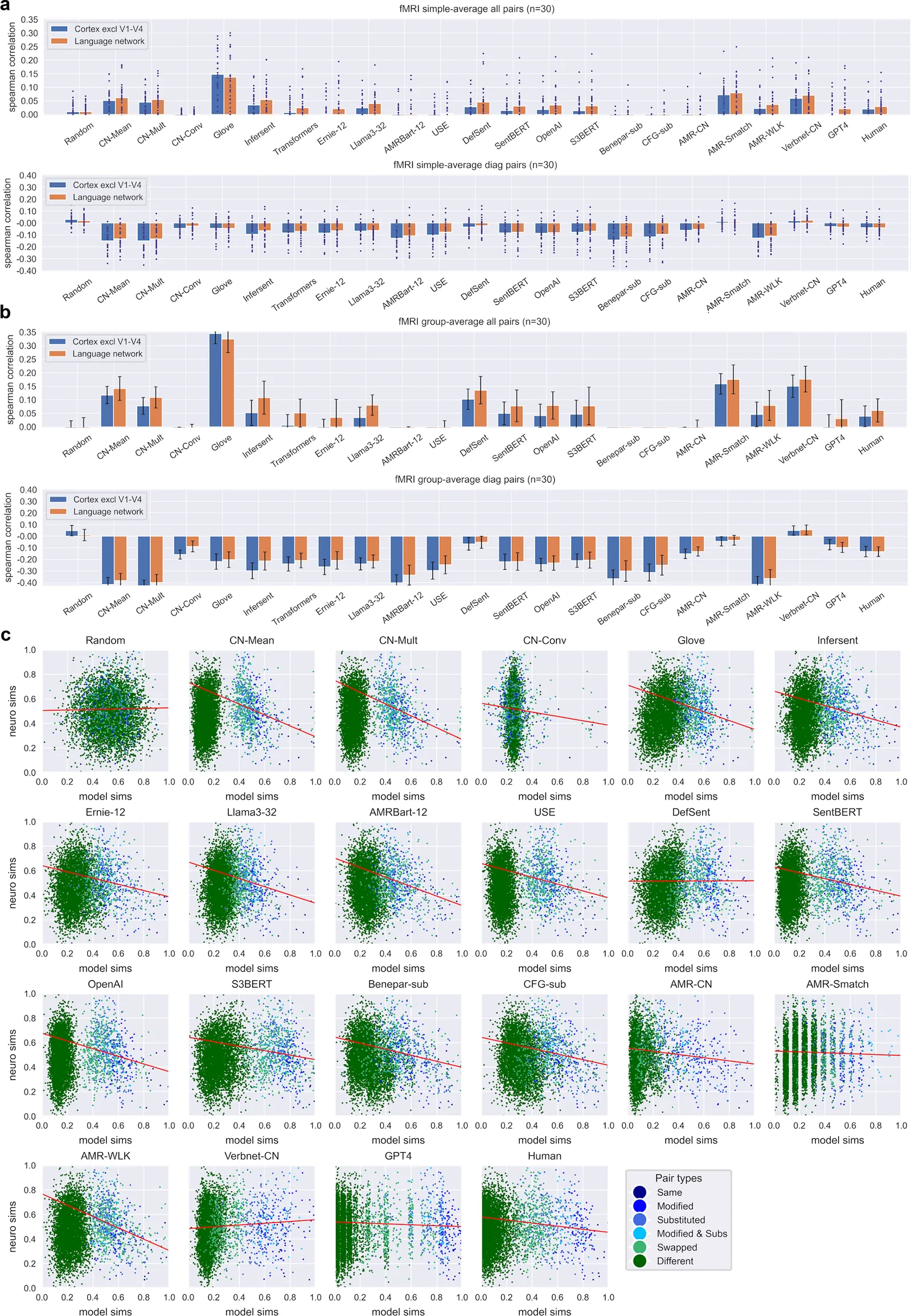
Correlations between all computational models and brain activity using dimension insensitive Euclidean metric (DIEM) similarity metric, with visual similarity control. Partial correlations between representational similarity analysis (RSA) matrices of all computational models and the brain RSA matrix, controlling for the RSA similarity of visual cortex activity averaged over participants. Blue bars show correlations computed over all stable (excluding visual regions V1–V4), while green bars show correlations for stable voxels in the language network. (**a**) Partial correlations for each individual participant shown as blue dots, with the simple average over individual correlations shown as a bar. (**b**) Partial correlations computed using the group-averaged RSA matrix. Error bars show 95% confidence intervals calculated by bootstrap resampling over participants. (**c**) Scatterplots showing the relationship between model similarities (horizontal axis) and group-average neural similarities (vertical axis) for all computational models. Each dot corresponds to a single pairwise similarity, scaled to between 0 and 1 for visualisation. While all sentence pairs are shown, regression lines (red) are computed over the block diagonal pairs only.

**Appendix 1—figure 17.**
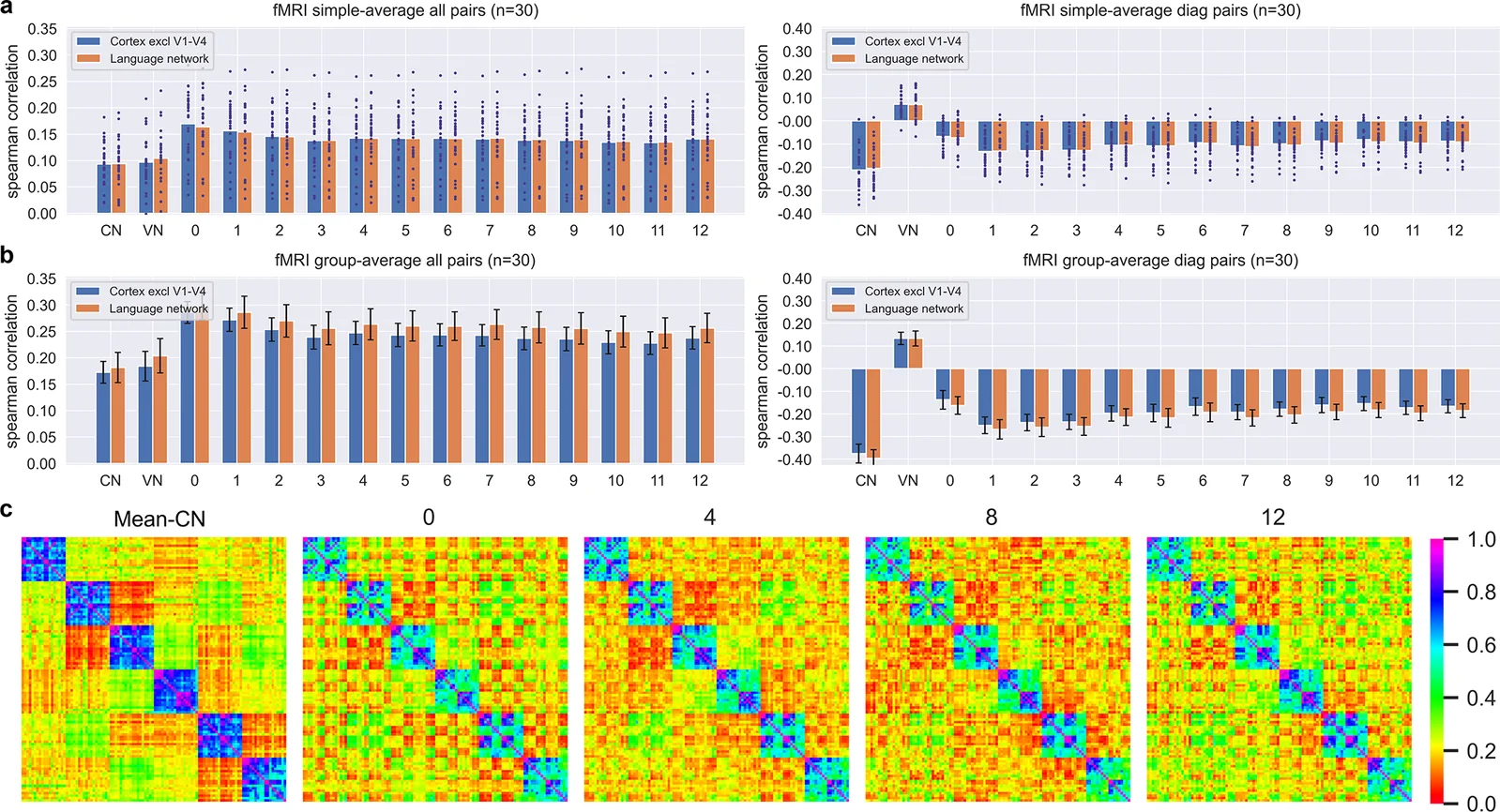
Average correlations between representational similarity analysis (RSA) matrices of each layer of AMRBart and the brain RSA matrix of each participant. Mean-CN (Mn) and VerbNet-CN hybrid (VN) models are also shown for comparison. (**a**) Partial correlations for each individual participant shown as blue dots, with the simple average over individual correlations shown as a bar. (**b**) Partial correlations computed using the group-averaged RSA matrix. Error bars show 95% confidence intervals calculated by bootstrap resampling over participants. (**c**) RSA matrices for the Mean-CN and VerbNet-CN models, along with selected layers of the AMRBart.

**Appendix 1—figure 18.**
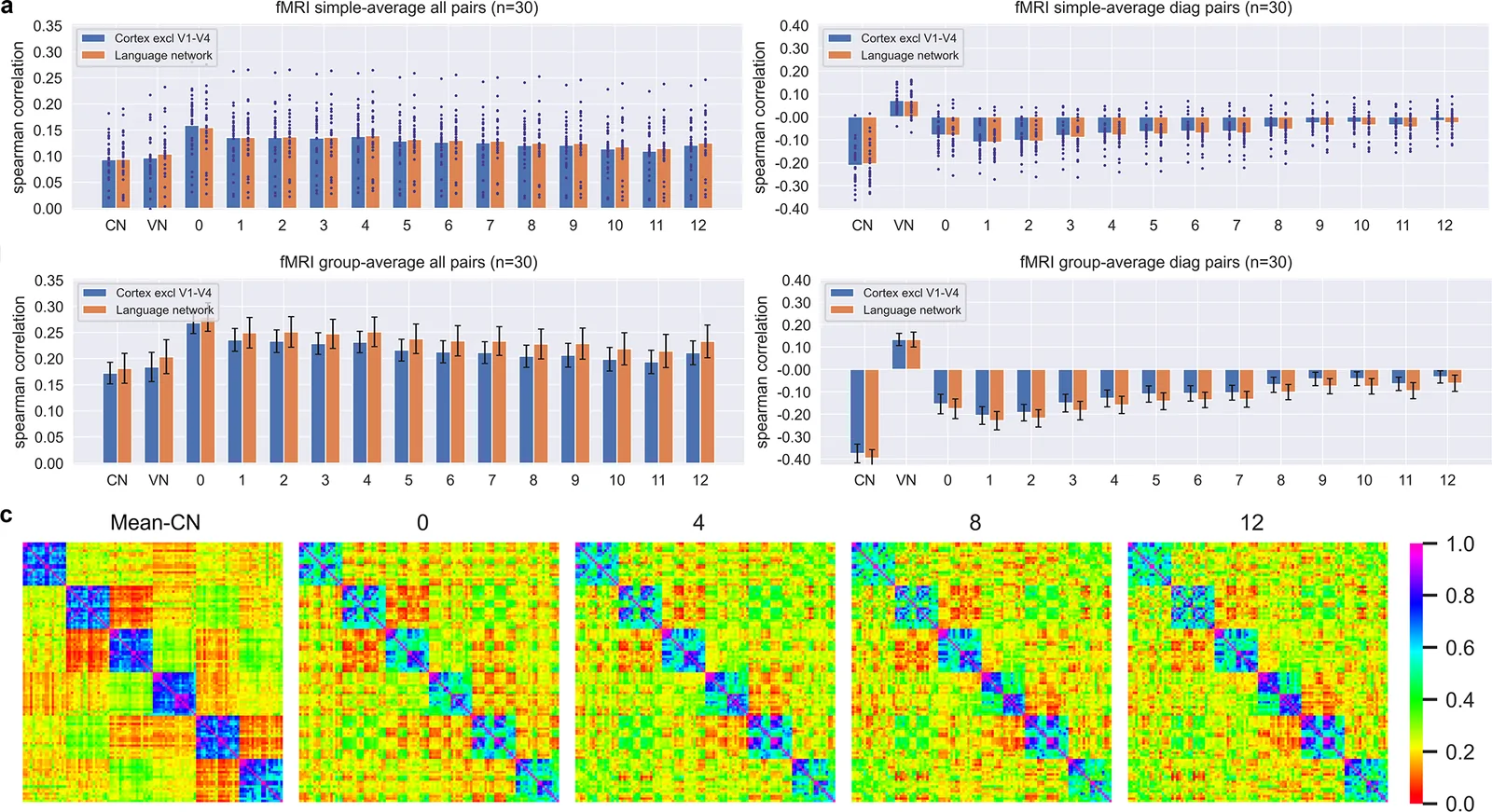
Average correlations between representational similarity analysis (RSA) matrices of each layer of ERNIE 2.0 and brain RSA matrix of each participant. Mean-CN (Mn) and VerbNet-CN hybrid (VN) models are also shown for comparison. (**a**) Partial correlations for each individual participant shown as blue dots, with the simple average over individual correlations shown as a bar. (**b**) Partial correlations computed using the group-averaged RSA matrix. Error bars show 95% confidence intervals calculated by bootstrap resampling over participants. (**c**) RSA matrices for the Mean-CN and VerbNet-CN models, along with selected layers of the ERNIE 2.0.

**Appendix 1—figure 19.**
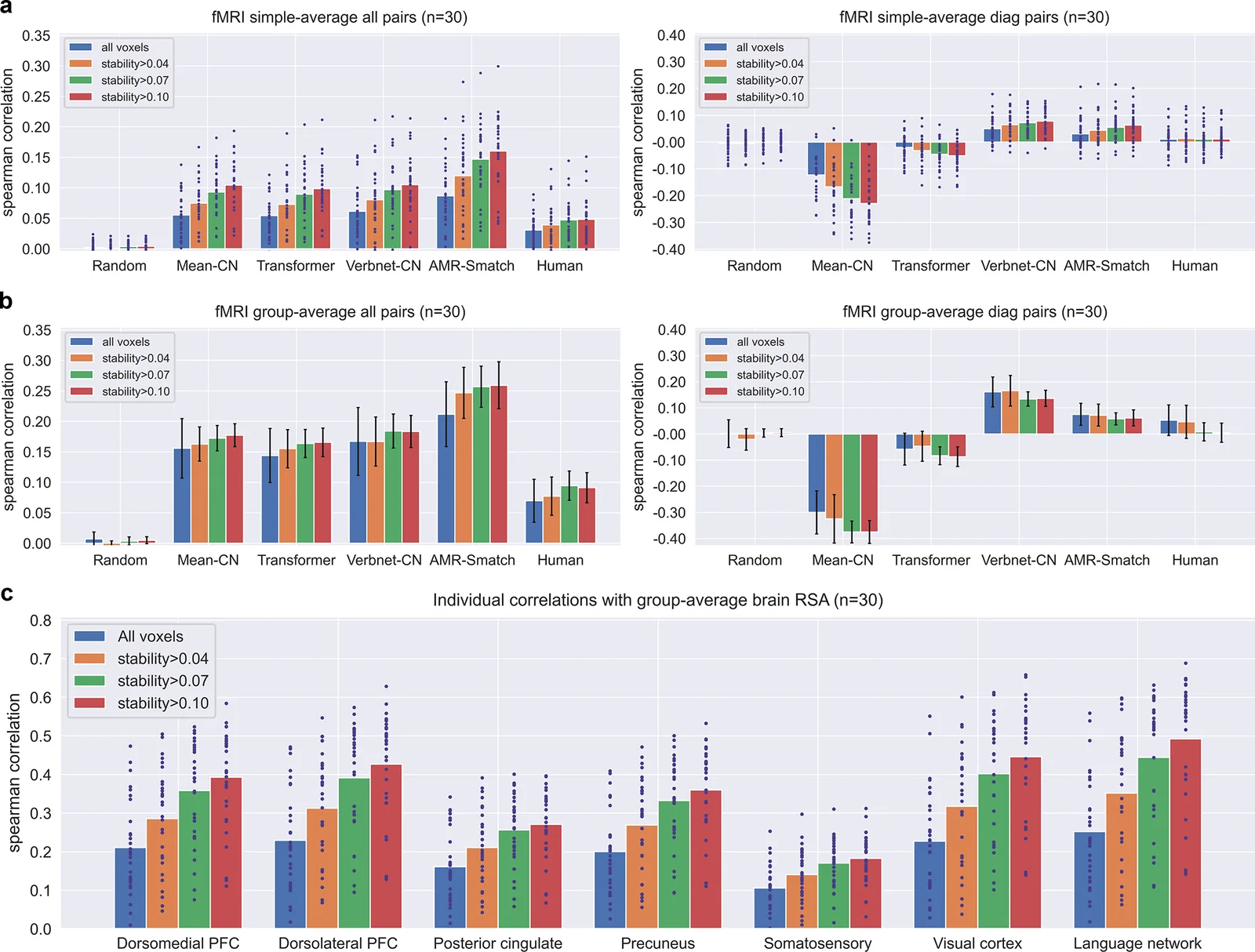
Comparison of model correlations with brain activity when varying the stability score threshold. Partial correlations between representational similarity analysis (RSA) matrices of five computational models and the brain RSA matrix, controlling for differences in sentence length. Bars are colour-coded based on the threshold used for voxel inclusion. (**a**) Partial correlations for each individual participant shown as blue dots, with the simple average over individual correlations shown as a bar. (**b**) Partial correlations computed using the group-averaged RSA matrix. Error bars show 95% confidence intervals calculated by bootstrap resampling over participants. (**c**) Correlations between RSA matrix of each individual participant and the group-averaged RSA matrix, organised by the voxel selection method used for computing the RSA matrix. Each dot corresponds to the correlation for a single participant.

**Appendix 1—figure 20.**
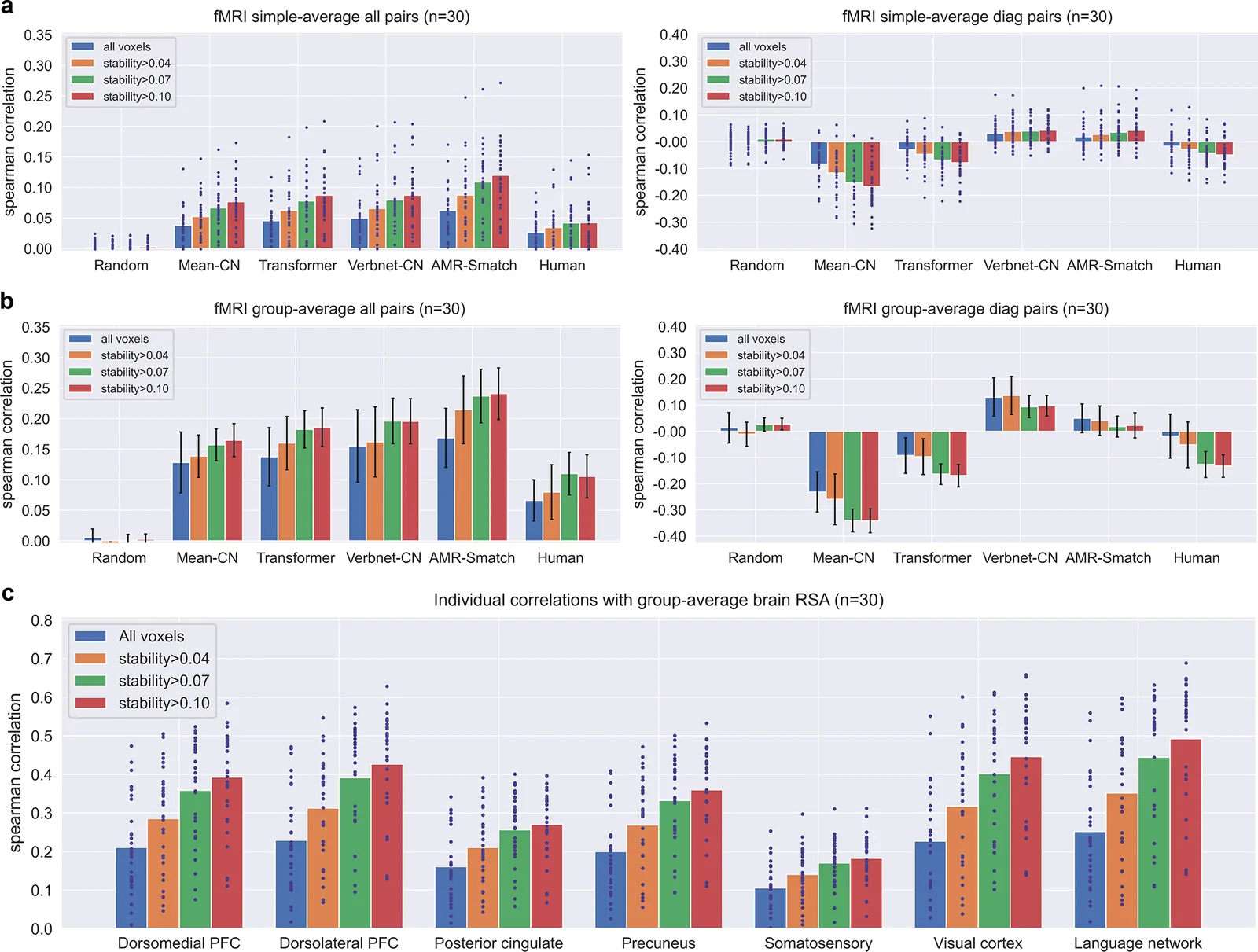
Comparison of model correlations with brain activity when varying the stability score threshold. Partial correlations between representational similarity analysis (RSA) matrices of five computational models and the brain RSA matrix, controlling for differences in sentence length and minimum sentence length. Bars are colour-coded based on the threshold used for voxel inclusion. (**a**) Partial correlations for each individual participant shown as blue dots, with the simple average over individual correlations shown as a bar. (**b**) Partial correlations computed using the group-averaged RSA matrix. Error bars show 95% confidence intervals calculated by bootstrap resampling over participants. (**c**) Correlations between RSA matrix of each individual participant and the group-averaged RSA matrix, organised by the voxel selection method used for computing the RSA matrix. Each dot corresponds to the correlation for a single participant.

**Appendix 1—figure 21.**
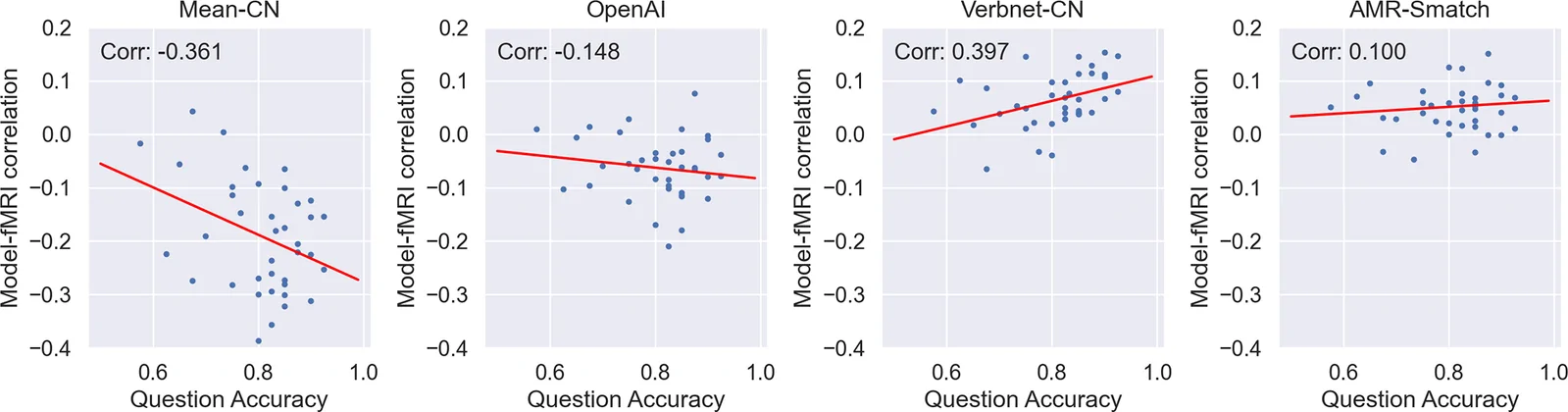
Correlation between participant accuracy in attention check questions and model similarity to brain representations. Scatterplot showing the accuracy score for each participant on attention check questions presented during the scanning run (horizontal axis) against the model correlation with all sentence pairs computed over all stable cortical voxels excluding V1–V4 (vertical axis). Each dot shows the results for a single participant, with all 38 participants included who achieved a score on the questions above chance level (25%).

**Appendix 1—figure 22.**
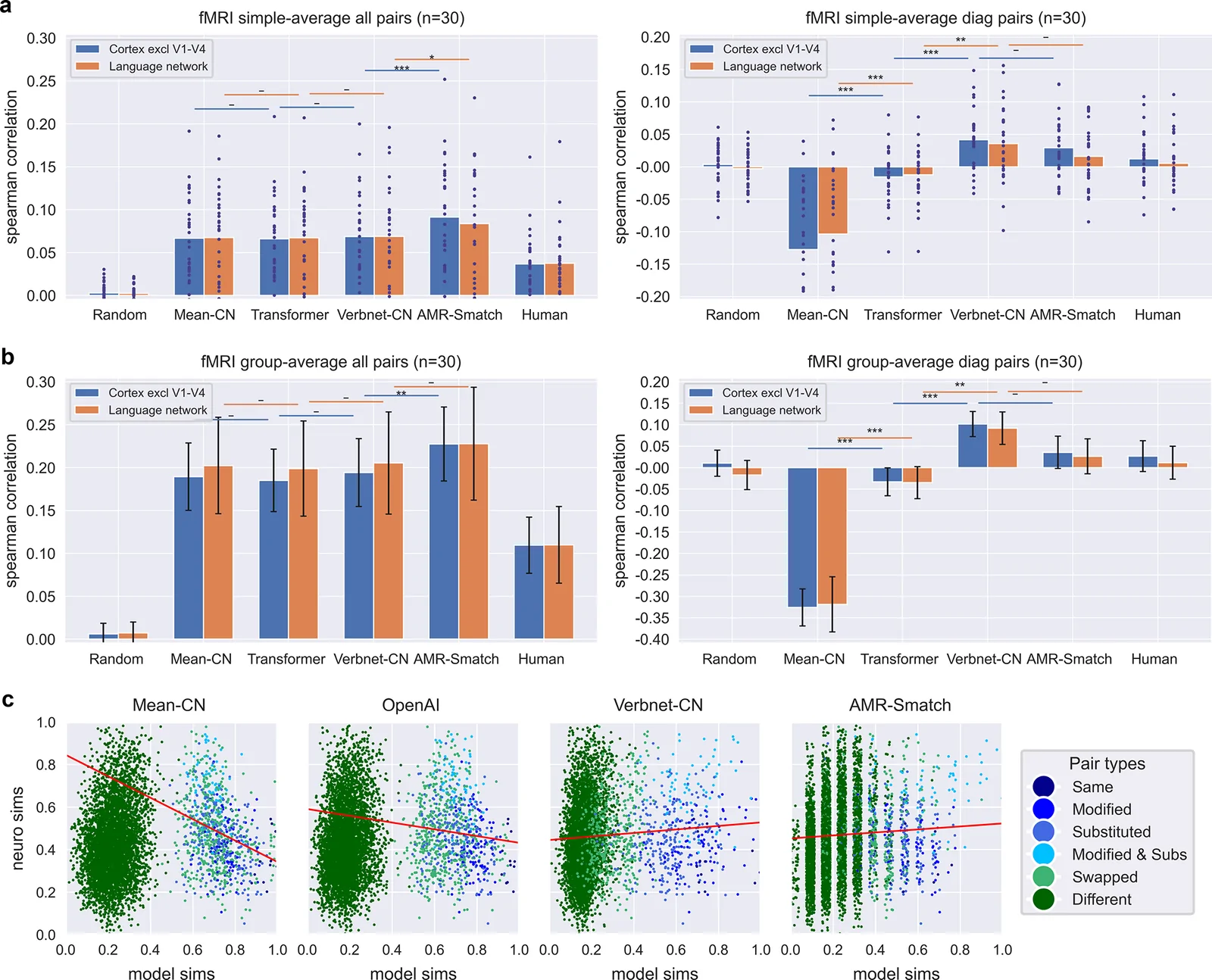
Model correlations with brain activity for all sentence pairs and the block diagonal subset of sentence pairs, using the final 3 s of each stimulus. Partial correlations between representational similarity analysis (RSA) matrices of five computational models (Random, Mean, Transformers, Hybrid, and Graph) and the brain RSA matrix, controlling for differences in sentence length and using the final 3 s for each stimulus. Blue bars show correlations computed over all stable (excluding visual regions V1–V4), while green bars show correlations for stable voxels in the language network. Notation for statistical significance: * for p<0.05, ** for p<0.01, and *** for p<0.001, with Bonferroni correction for three independent comparisons. (**a**) Partial correlations for each individual participant shown as blue dots, with the simple average over individual correlations shown as a bar. (**b**) Partial correlations computed using the group-averaged RSA matrix. Error bars show 95% confidence intervals calculated by bootstrap resampling over participants. (**c**) Scatterplots showing the relationship between model similarities (horizontal axis) and group-average neural similarities (vertical axis) for all four computational models. Each dot corresponds to a single pairwise similarity, scaled to between 0 and 1 for visualisation. While all sentence pairs are shown, regression lines (red) are computed over the block diagonal pairs only.

## Data Availability

Our fMRI dataset is available for download on OpenNeuro: https://openneuro.org/datasets/ds007393. All code used is available at https://github.com/Fods12/sentence-meaning-in-the-brain (copy archived Fodor, 2026). The following dataset was generated: FodorJ MurawskiC SuzukiS 2026Representations of Sentence Meaning in the Human Brain (RSMB) DatasetOpenNeurods007393

## Additional files

MDAR checklist

## Appendix 1

### Supplementary methods

#### Full fMRIprep methods

Results included in this manuscript come from preprocessing performed using *fMRIPrep* 23.2.3 (Esteban et al., 2019; Esteban et al., 2018), which is based on *Nipype* 1.8.6 (Gorgolewski et al., 2011; Gorgolewski et al., 2018). Many internal operations of *fMRIPrep* use *Nilearn* 0.10.2 (Abraham et al., 2014), mostly within the functional processing workflow. For more details of the pipeline, see the section corresponding to workflows in fMRIPrep’s documentation.

##### Preprocessing of B0 inhomogeneity mappings

A total of eight fieldmaps were found available within the input BIDS structure for this particular subject. A *B0*-nonuniformity map (or *fieldmap*) was estimated based on two (or more) EPI references with topup (Andersson et al., 2003).

##### Anatomical data preprocessing

A total of one T1-weighted (T1w) image was found within the input BIDS dataset. The T1w image was corrected for intensity non-uniformity with N4BiasFieldCorrection (Tustison et al., 2010), distributed with ANTs 2.5.0 (Avants et al., 2008), and used as T1w reference throughout the workflow. The T1w reference was then skull-stripped with a *Nipype* implementation of the antsBrainExtraction.sh workflow (from ANTs), using OASIS30ANTs as target template. Brain tissue segmentation of cerebrospinal fluid (CSF), white matter (WM), and grey matter (GM) was performed on the brain-extracted T1w using fast FSL fsl_fast. Brain surfaces were reconstructed using recon-all FreeSurfer 7.3.2 (Dale et al., 1999), and the brain mask estimated previously was refined with a custom variation of the method to reconcile ANTs-derived and FreeSurfer-derived segmentations of the cortical GM of Mindboggle (Klein et al., 2017). Volume-based spatial normalisation to one standard space (MNI152NLin2009cAsym) was performed through nonlinear registration with antsRegistration ANTs 2.5.0, using brain-extracted versions of both T1w reference and the T1w template. The following template was selected for spatial normalisation and accessed with *TemplateFlow* (Ciric et al., 2022): *ICBM 152 Nonlinear Asymmetrical template version 2009c* (Fonov et al., 2009), TemplateFlow ID: MNI152NLin2009cAsym.

##### Functional data preprocessing

For each of the eight BOLD runs found per subject (across all tasks and sessions), the following preprocessing was performed. First, a reference volume was generated, using a custom methodology of *fMRIPrep*, for use in head motion correction. Head-motion parameters with respect to the BOLD reference (transformation matrices and six corresponding rotation and translation parameters) are estimated before any spatiotemporal filtering using mcflirt FSL (Jenkinson et al., 2002). The estimated *fieldmap* was then aligned with rigid-registration to the target EPI reference run. The field coefficients were mapped on to the reference EPI using the transform. The BOLD reference was then co-registered to the T1w reference using bbregister (FreeSurfer), which implements boundary-based registration (Greve and Fischl, 2009). Co-registration was configured with six degrees of freedom. Several confounding time series were calculated based on the *preprocessed BOLD*: framewise displacement (FD), DVARS, and three region-wise global signals. FD was computed using two formulations following Power (absolute sum of relative motions; Power et al., 2014) and Jenkinson (relative root mean square displacement between affines; Jenkinson et al., 2002). FD and DVARS are calculated for each functional run, both using their implementations in *Nipype* following published definitions (Power et al., 2014). The three global signals are extracted within the CSF, the WM, and the whole-brain masks. Additionally, a set of physiological regressors were extracted to allow for component-based noise correction *CompCor* (Behzadi et al., 2007).

Principal components are estimated after high-pass filtering the *preprocessed BOLD* time series (using a discrete cosine filter with 128 s cut-off) for the two *CompCor* variants: temporal (tCompCor) and anatomical (aCompCor). tCompCor components are then calculated from the top 2% variable voxels within the brain mask. For aCompCor, three probabilistic masks (CSF, WM, and combined CSF+WM) are generated in anatomical space. The implementation differs from that of Behzadi et al. in that instead of eroding the masks by 2 pixels on BOLD space, a mask of pixels that likely contain a volume fraction of GM is subtracted from the aCompCor masks. This mask is obtained by dilating a GM mask extracted from the FreeSurfer’s *aseg* segmentation, and it ensures components are not extracted from voxels containing a minimal fraction of GM. Finally, these masks are resampled into BOLD space and binarized by thresholding at 0.99 (as in the original implementation). Components are also calculated separately within the WM and CSF masks. For each CompCor decomposition, the *k* components with the largest singular values are retained, such that the retained components’ time series are sufficient to explain 50% of variance across the nuisance mask (CSF, WM, combined, or temporal). The remaining components are dropped from consideration.

The head-motion estimates calculated in the correction step were also placed within the corresponding confounds file. The confound time series derived from head motion estimates and global signals were expanded with the inclusion of temporal derivatives and quadratic terms for each (Satterthwaite et al., 2013). Frames that exceeded a threshold of 0.5 mm FD or 1.5 standardized DVARS were annotated as motion outliers. Additional nuisance time series are calculated by means of principal components analysis of the signal found within a thin band (*crown*) of voxels around the edge of the brain, as proposed previously (Patriat et al., 2017). All resamplings can be performed with *a single interpolation step* by composing all the pertinent transformations (i.e. head-motion transform matrices, susceptibility distortion correction when available, and co-registrations to anatomical and output spaces). Gridded (volumetric) resamplings were performed using nitransforms, configured with cubic B-spline interpolation.

#### Instructions for behavioural task

The following instructions were presented to participants in the online Prolific experiment:

Please read the following instructions carefully before proceeding.
In this questionnaire you will be presented with 112 paired sentences, in 16 groups of 7 questions per page. Your task is to judge how similar is the meaning of the two sentences. You will make this judgement by choosing a rating from 1 (very dissimilar) to 7 (very similar). In providing your rating, consider both the similarity in meaning of the individual words contained in the sentences, as well as the similarity of the overall idea or meaning expressed by the sentences.
Some of the sentences may be slightly unusual or ambiguous; nevertheless you should do your best to understand their likely meaning. Bear in mind that we are not looking for any one specific ‘right answer’ or strategy in your responses. Your task is simply to make a judgement about how similar you think is the meaning of the two paired sentences. The only exception is that if you find a sentence that truly does not make any sense at all, then you should give it a very low similarity to whatever it is paired with. In all other cases, make your best judgement based on your assessment of overall meaning of the sentences.
There is no time limit to this task; however, each sentence pair should not take more than a few seconds to judge. There is no need to spend a long time pondering each sentence. In total the task should take around 20–30 min.
Thanks very much for your time!

### Supplementary tables

#### Complete set of stimulus sentences

**Table:**

Set	Type	Sentence
1	Base	The cameraman brought the equipment to the director.
1	Same	The cameraman brought the new equipment to the director.
1	Substituted	A painter brought the equipment to the director.
1	Substituted	The cameraman sold the equipment to the director.
1	Substituted	The cameraman brought a hot lunch for the director.
1	Modified	Every morning the cameraman brought the equipment to the impatient director.
1	Modified	A team of cameramen brought all the necessary equipment to the busy director.
1	Modified	The cameraman begrudgingly brought the awkward equipment up from the basement to the director.
1	Modified	At dawn the industrious cameraman brought the essential equipment to the director in his downtown office.
1	Swapped	The director brought the cameraman to the equipment.
1	Swapped	The director brought the cameraman to his equipment.
1	Swapped	The director brought his cameraman a hot lunch.
1	Swapped	The director sold the cameraman’s equipment.
1	Swapped	The director brought the painter to his equipment.
1	Swapped	Every morning the director brought the cameraman to the expensive equipment.
1	Swapped	The director inexplicably brought the cameraman to the useless old equipment in the storeroom.
1	Swapped	The renowned director brought the impatient cameraman to the equipment from his last project.
1	Swapped	On the weekend the helpful film director brought the inexperienced cameraman to his equipment in the storeroom.
2	Base	The psychologist spoke to the secretary about the patient.
2	Same	The psychologist often spoke to the secretary about the patient.
2	Substituted	The cleaner spoke to the secretary about the patient.
2	Substituted	The psychologist warned the secretary about the patient.
2	Substituted	A psychologist spoke to her gardener about the patient.
2	Modified	The psychologist sometimes spoke to her secretary about the problematic patient.
2	Modified	The distinguished psychologist spoke in her office to the diligent secretary about her patients.
2	Modified	Occasionally the psychologist accidentally spoke to his secretary about one patient within earshot of other patients.
2	Modified	One per week the psychologist spoke delicately to her experienced secretary about her most difficult patients.
2	Swapped	The secretary spoke to the patient about the psychologist.
2	Swapped	The secretary often spoke to the patient about the psychologist.
2	Swapped	The gardener spoke to the patient about her psychologist.
2	Swapped	The secretary recommended the patient to the psychologist.
2	Swapped	The secretary spoke to the patient about the cleaner.
2	Swapped	The secretary sometimes spoke rudely to patients about the psychologist.
2	Swapped	The secretary at the desk spoke clearly with the disabled patient about their next psychologist appointment.
2	Swapped	The exhausted secretary spoke slowly to the patient about their psychologist late in the evening.
2	Swapped	The concerned secretary spoke gently to the anxious patient in a private consultation room about the psychologist.
3	Base	The company purchased the apartment block from the family.
3	Same	The company bought the apartment block from the family.
3	Substituted	The government purchased the apartment block from the family.
3	Substituted	The company leased the apartment block to the family.
3	Substituted	The company purchased a valuable painting from the family.
3	Modified	In a calculated manoeuvre the company swiftly purchased the apartment block from the family.
3	Modified	A small company purchased the drab concrete apartment block from the family for later redevelopment.
3	Modified	Shortly before the election the company unwittingly purchased a defective apartment block from the family.
3	Modified	The foreign company purchased a decrepit apartment block from the wealthy family for an outrageous price.
3	Swapped	The family blocked the purchase of the apartment by the company.
3	Swapped	The family blocked the purchase of the apartment building by the company.
3	Swapped	The family blocked the purchase of their vintage car by the company.
3	Swapped	The family secured the purchase of their apartment by the company.
3	Swapped	The government blocked the purchase of the apartment by the company.
3	Swapped	The stubborn family blocked the purchase of the apartment by the company for six months.
3	Swapped	The family from across town blocked the purchase of the modern apartment by the unpopular company.
3	Swapped	After a long court battle the family finally blocked the purchase of the apartment by the unscrupulous corporation.
3	Swapped	For no good reason the family blocked the purchase of an old apartment by the local company for six months.
4	Base	The dog smelled the strange object near the children.
4	Same	The dog sniffed the strange object near the children.
4	Substituted	The cat smelled the strange object near the children.
4	Substituted	The dog noticed the strange object near the children.
4	Substituted	The dog smelled smoke near the children.
4	Modified	The dog cautiously sniffed the mysterious object adjacent to the group of children.
4	Modified	The cute little dog smelled a strange object near the young children in the grass.
4	Modified	The feral dog smelled a strange rusty object near the children at the beach.
4	Modified	With its heightened sensory perception the dog smelled an unusual object near the large group of children at the school.
4	Swapped	The children objected to the unpleasant smell of the dog.
4	Swapped	The children objected to the dog’s disagreeable smell.
4	Swapped	The father objected to the unpleasant smell of the dog.
4	Swapped	The children noticed the unpleasant smell of the dog.
4	Swapped	The children objected to the unpleasant smell of the cat.
4	Swapped	The children rarely objected to the unpleasant smell of their neighbour’s dog.
4	Swapped	The group of children objected to the unpleasant smell of the large black dog from the park.
4	Swapped	The uncaring children objected to the smell of the sick dog at the vet.
4	Swapped	After five long days the disappointed children finally objected to the unpleasant smell of their favourite new dog.
5	Base	The lobbyist wrote to the politician about the reporter.
5	Same	The lobbyist wrote to the politician about the journalist.
5	Substituted	The nonprofit organisation wrote to the politician about the reporter.
5	Substituted	The lobbyist warned the politician about the reporter.
5	Substituted	The lobbyist wrote to the newspaper about the reporter.
5	Modified	In response to recent events the lobbyist quickly wrote to the politician about the reporter.
5	Modified	Over the weekend the lobbyist wrote a comprehensive report to the politician about the disgraced reporter.
5	Modified	The experienced lobbyist wrote a painstakingly detailed email to the politician about the reporter’s recent activities.
5	Modified	The lobbyist composed an eloquent message to the politician on behalf of his client about the reporter.
5	Swapped	The reporter wrote to the lobbyist about the politician.
5	Swapped	The journalist wrote to the lobbyist about the politician.
5	Swapped	The academic wrote to the lobbyist about the politician.
5	Swapped	The reporter interviewed the lobbyist about the politician.
5	Swapped	The reporter wrote to the nonprofit organisation about the politician.
5	Swapped	On her new laptop the reporter wrote a short blog post for the lobbyist regarding the politician.
5	Swapped	The reporter regularly wrote to the lobbyist about the powerful politician via an encrypted message system.
5	Swapped	Using pen and paper the reporter meticulously wrote a personal letter to the lobbyist about the politician.
5	Swapped	Out of concern for the public, the reporter wrote to the lobbyist about the politician’s recent decisions.
6	Base	The shopkeeper displayed the watch for customers.
6	Same	The shopkeeper displayed the watch for his customers.
6	Substituted	The salesperson displayed the watch for customers.
6	Substituted	The shopkeeper marketed the watch to customers.
6	Substituted	The shopkeeper displayed books for customers.
6	Modified	Each day the shopkeeper displayed the watch to customers in his store.
6	Modified	During busy periods the shopkeeper prominently displayed the watch for customers.
6	Modified	The incompetent shopkeeper foolishly displayed the watch to customers in an obscure location.
6	Modified	As a marketing technique the shopkeeper prominently placed the watch on a dedicated stand for customers.
6	Swapped	The customer watched over the display for the shopkeeper.
6	Swapped	A customer watched over the display for the shopkeeper.
6	Swapped	The surveillance camera watched over the display for the shopkeeper.
6	Swapped	The customer critiqued the display for the shopkeeper.
6	Swapped	The customer watched over the model for the shopkeeper.
6	Swapped	Every day the customer watched over the display for the shopkeeper during lunch.
6	Swapped	The customer patiently watched over the well-organised display for the shopkeeper for hours on end.
6	Swapped	The unscrupulous customer greedily watched the display behind the shopkeeper’s back during the busiest time of day.
6	Swapped	In the back room the nosey customer watched over the goods display for the shopkeeper on the security monitor.
