# Supplementary material for: Large language models poorly capture how word order shapes representations of sentence meaning in the human brain
Source: eLife. 2026 Oct 23;14:RP108442. doi: 10.7554/eLife.108442 (PMC13694100; doi:10.7554/eLife.108442)
Supplement: Peer review history

# Editor report: eLife Assessment

**DOI:** 10.7554/eLife.108442.4.sa0

**Authors:** Nai Ding

**Affiliations:** Zhejiang University China

**Keywords:** Solid, Valuable

The paper presents a **valuable** finding that the human brain and models that incorporate sentence structures can capture sentence-level semantics beyond word meaning, while large language models behave differently. The evidence supporting the authors' claims is **solid**, though the stimuli are highly controlled and some analyses could be more thorough. This work will be of interest to researchers in language neuroscience and those developing language models.

# Referee report: Reviewer #3 (Public review)

**DOI:** 10.7554/eLife.108442.4.sa1

Summary:

Large Language Models have revolutionized Artificial Intelligence and can now match or surpass human language abilities on many tasks. This has fueled interest in cognitive neuroscience in exposing representational similarities between Language Models and brain recordings of language comprehension. The current study breaks from this mold by: (1) Systematically identifying sentence structures for which brain and Large Language Model representations diverge. (2) Using models structured by semantic roles to help account for divergences. As such the study may now fuel interest in characterizing how Large Language Models and brain representations differ, which may prompt new more brain like language models.

Strengths:

(1) This study challenges a literature trend that has touted similarities between Transformer models and human cognition based on representational correlations with brain activity. This challenge is substantiated by identifying sentences for which brain and model representations of sentences diverge.

(2) This study conducts a rigorous pre-registered analysis of a comprehensive selection of the state-of-the-art Large Language Models, on a controlled sentence comprehension fMRI dataset. The analysis is conducted within a Representation Similarity framework to support similarity comparisons between graph structures and brain activity without needing to vectorize graphs. Transformer language models are predicted and shown to diverge from brain representations on subsets of sentences with similar word-level content but different sentence structures.

(3) The study introduces a 7T fMRI sentence comprehension dataset and accompanying human sentence similarity ratings which may be a fruitful resource for developing more human-like language models. Unlike other model-based sentence datasets, the relation between grammatical structure and word-level content is controlled, and subsets of sentences for which models and brains diverge are identified.

Weaknesses:

(1) The interpretation of findings is nuanced. Although Transformers underperform as brain models on the critical subsets of controlled sentences, a Transformer outperforms all other models when evaluated on the union of all sentences when both word-level content and structure vary. Transformers also yield equivalent or better models of human behavioral data. Thus, although Transformers have demonstrable flaws as human models which are pinpointed here, when tested on all sentences (some) Transformers are more human-like than the other models considered.

(2) There may be confounds between the critical sentence structure manipulations and visual processing. This is inconvenient because activation in brain regions that process semantics tends to partially correlate with low-level representations of sentence surface features encoded in visual cortex. Although the study commendably controls for confounds associated with sentence length, correlations with key sentence structure models are most salient in visual cortex and diminish in other brain networks when V1-V4 activation is controlled for.

(3) Sentence similarity computations are emphasized as the basis for unifying comparative analyses of graph structures and vector data. A strength of this approach is that correlation is not always the ideal similarity metric. However, a weakness is that similarity computations are not unified across models. This has practical consequences because different similarity metrics applied to the same model produce positive or negative correlations with brain data.

Comments on revised version.

Thanks again for the responses. In particular, the new sentence-length control is helpful for interpreting the anomalous outcomes from the DIEM analysis, and the amendments to the discussion are appreciated.

# Author comment: Author response

**DOI:** 10.7554/eLife.108442.4.sa2

**Authors:** James Fodor, Carsten Murawski, Shinsuke Suzuki

**Affiliations:** The University of Melbourne Melbourne Australia; The University of Melbourne Melbourne Australia; Tohoku University Sendai Japan

The following is the authors’ response to the previous reviews.

**Public Reviews:**

**Reviewer #1 (Public review):**

(1) The rationale behind averaging sentence embeddings across multiple transformer models (with different architectures and training objectives) is unclear. These transformer-based models have different training paradigms and model architectures, which may result in misaligned semantic spaces. The averaging operation may dilute the distinct sentence representations learned by each model, potentially weakening the overall semantic encoding for sentences. Please clarify this choice or cite supporting methodology.

The reviewer questions the rationale for averaging sentence embeddings across different models. However, our method involves computing correlations separately for each model, then averaging the correlations. We apologize for the confusion. We have clarified this on page 3:

“Results for the ‘Transformers’ model are computed by computing correlations separately for five different transformer models and then taking a simple average of these correlations. Results for each individual transformer are presented in Supplementary Information Figure S2.”

(2) All structure-sensitive models discussed incorporate semantics to some extent. Including a purely syntactic baseline, such as a model based on context-free grammar, would help confirm the importance of syntactic structures.

Following the suggestion, we have implemented two syntactic models and discuss the results on page 10:

“We also found that purely syntactic models based on constituency parses (see Benepar and CFG) show poor correlations with brain activity (see Supplementary Information Figure S2). Examining the corresponding RSA matrices (see Figure S1), this seems to be due to such models being overly sensitive to syntactic form, and relatively insensitive to which words are assigned to different nodes within the syntactic tree. This is most evident for the edit-distance similarity metric, and to a lesser extent also for the subtree similarity metric. This finding highlights the value of hybrid approaches designed to appropriately balance sensitivity to lexical, syntactic, and compositional information in representing semantic information at the sentence level.”

(3) In Figure 2, human behavioral judgments show weak correlations with neural data, and even fall below those of computational models, suggesting the behavioral judgments may not reflect the sentence structures in a brain-like way. This discrepancy between behavioral and neural data should be clarified, as it affects the interpretation of the results.

While the behavioural judgements are made by different participants and involve a different task than the neuroimaging results, nonetheless we agree the difference is surprising and warrants more detailed consideration. We have included a more detailed discussion of this issue on page 11:

“Our study has several limitations. First, we found a surprisingly low correlation between behavioural ratings and brain activations (see Figure 2). This may be partly explained by differences in task structure. In the behavioural experiment, participants viewed many pairs of related sentences, and were explicitly asked to pay attention to differences in the words of each sentence. In contrast, in the fMRI task, participants read one sentence at a time without an explicit comparison. In addition, we suspect that presentation of so many sentence pairs with highly similar structures may have biased the way in which participants rated sentence similarity. Modifications to the behavioural task to mitigate these aspects may reduce the divergence between behavioural and brain findings.”

(4) To better contextualize model and neural performance, sentence similarity should be anchored to a notion of semantic "ground truth", such as the matrix shown in Figure 1a. Comparing this reference with human judgments, brain responses, and model similarities would help establish an upper bound.

While our design matrix served as the basis for constructing a set of stimuli with systematic modifications, we respectfully suggest that it should not be regarded as a ‘semantic ground truth’. Sentence pairs within each category will not have the same degrees of semantic similarity since the words and context differ across sentences in a graded manner. Furthermore, while we anticipated ‘different’ sentence pairs would be less similar than ‘swapped’ sentence pairs, and that within each of the six block diagonals the ‘modified’ or ‘substituted’ sentence pairs would be the most similar, we did not have any prediction about the magnitude of these differences. Our goal was to construct a set of sentence pairs which spanned a range of semantic similarities, and allowed for dissociation between lexical similarity and overall similarity in meaning. The design matrix is not intended to represent a ‘ground truth’ that human judgements or brain representations would be expected to conform with.

(5) The structure of this paper is confusing. For instance, Figure 5 is cited early but appears much later. Reordering sections and figures would enhance readability.

We agree that placement of figures was not ideal in the previous draft. We have reworked the manuscript so that all figures appear closer to their mention in the text, and the figure (now Figure 3) appears in the correct order. We have also substantially revised the discussion, and included subheadings to help guide the reader through the various different issues we include.

(6) While the analysis is broad and comprehensive, it lacks depth in some respects. For instance, it remains unclear what specific insights are gained from comparing across brain regions (e.g., whole brain, language network, and other subregions). Similarly, the results of simple-average and group-average RSA appear quite similar and may not advance the interpretation.

We included both analyses in line with our preregistration, and also because we believe the fact that two distinct approaches to analyzing the data yield similar results strengthens our conclusions.

(7) While explaining the grid-like pattern due to sentence length is important, this part feels somewhat disconnected from the central question of this paper (word order). It might be better placed in supplementary material.

We believe that the grid-like pattern in the RSA results is an important unexpected finding that warrants discussion in the main manuscript.

**Reviewer #3 (Public review):**

(1) The interpretation of findings is nuanced. Although Transformers underperform as brain models on the critical subsets of controlled sentences, a Transformer outperforms all other models when evaluated on the union of all sentences when both word-level content and structure vary. Transformers also yield equivalent or better models of human behavioral data. Thus, although Transformers have demonstrable flaws as human models, which are pinpointed here, in the general case, (some) Transformers are more human-like than the other models considered.

The reviewer argues that we overstate some of our conclusions, as several transformers achieve higher brain correlations than the hybrid model when computed over all sentence pairs, as well as on the behavioural data. In response, we first note that our primary interest in this paper is on the block-diagonal sentence pairs, as these were specifically designed to interrogate how different models represent sentence structure. The comparison with all sentence pairs is presented for comparison but is not our primary focus on this paper, as also reflected in the pre-registered prediction that our VerbNet-CN hybrid model would show higher brain correlations than transformers over this block diagonal subset.

Second, we have included a new analysis in the revised manuscript (Figure S9) where we compute brain correlations controlling for the pattern of similarities observed in the primary visual cortex (averaged over participants), as a way to control for visual similarity. This added control substantially reduces the brain correlations of the transformers, such that they all have lower correlations than VerbNet-CN and AMR-smatch even over the set of all sentence pairs. We provide interpretation of this result in the discussion.

Third, we would like to note one of the disadvantages of transformers as a model of mind or brain representations is that they are largely a ‘black box’ whose workings are poorly understood. One advantage of hybrid models like our simple semantic role model is that they can be much easier to interpret, thereby enabling them to be used to determine which features are most important for brain representations of sentence meaning, and what mechanisms are used to combine individual words into a full sentence. Given their relative simplicity and interpretability, we believe hybrid models have considerable value as scientific tools, even in cases where they achieve comparable correlations to transformers. We have added a short discussion of this issue in the revised manuscript (page 10).

(2) There may be confounds between the critical sentence structure manipulations and visual representations of sentence stimuli. This is inconvenient because activation in brain regions that process semantics tends to partially correlate with visual cortex representations, and computational models tend to reflect the number of words/tokens/elements in sentences. Although the study commendably controls for confounds associated with sentence length, there could still be residual effects that remain. For instance, the Graph model correlates most strongly with the visual cortex despite these sentence length controls.

We agree with the reviewer that this is a potential confound. As noted in the previous response, we have implemented a new control analysis in which we directly control for visual similarities as reflected in participant-averaged similarities of primary visual cortex activations in response to all stimuli. These results are shown in Figures S8-S11 in the SI. We show that transformer correlations are reduced much more than graph and hybrid models with this control. Also, we note that the AMR-smatch graph model shows high correlations with other brain regions even after removing correlations with the visual cortex (Figure S10). This indicates that the model represents a range of sentence features, including both superficial visual or length-related features, as well as semantic features that are represented in common with language and other cortical regions.

(3) Sentence similarity computations are emphasized as the basis for unifying comparative analyses of graph structures and vector data. A strength of this approach is that correlation is not always the ideal similarity metric. However, a weakness is that similarity computations are not unified across models. This has practical consequences here because different similarity metrics applied to the same model produce positive or negative correlations with brain data.

The reviewer notes that the method for computing similarities differs between the vector-based (mean and transformer) models, and the hybrid and syntax-based models, thereby potentially adding an additional confound to our results. We agree that this is a potential limitation, and our correlations should always be understood as applying to a model paired with a similarity metric. However, we believe that this is mostly unavoidable when comparing different formalisms. In the revised manuscript we have incorporated an entirely new similarity metric for vector-based models (DIEM similarity), as well as an extended discussion of the effect of different similarity metrics for graph and hybrid models.
